# Peak Scores Significantly Depend on the Relationships between Contextual Signals in ChIP-Seq Peaks

**DOI:** 10.3390/ijms25021011

**Published:** 2024-01-13

**Authors:** Oleg V. Vishnevsky, Andrey V. Bocharnikov, Elena V. Ignatieva

**Affiliations:** 1 Institute of Cytology and Genetics, 630090 Novosibirsk, Russia; eignat@bionet.nsc.ru; 2 Department of Natural Science, Novosibirsk State University, 630090 Novosibirsk, Russia; andrey.bocharnikov@gmail.com

**Keywords:** chromatin immunoprecipitation with massively parallel sequencing, transcription factor binding sites, IUPAC motifs, co-binding of transcription factors, composite elements, multiple regression

## Abstract

Chromatin immunoprecipitation followed by massively parallel DNA sequencing (ChIP-seq) is a central genome-wide method for in vivo analyses of DNA-protein interactions in various cellular conditions. Numerous studies have demonstrated the complex contextual organization of ChIP-seq peak sequences and the presence of binding sites for transcription factors in them. We assessed the dependence of the ChIP-seq peak score on the presence of different contextual signals in the peak sequences by analyzing these sequences from several ChIP-seq experiments using our fully enumerative GPU-based de novo motif discovery method, Argo_CUDA. Analysis revealed sets of significant IUPAC motifs corresponding to the binding sites of the target and partner transcription factors. For these ChIP-seq experiments, multiple regression models were constructed, demonstrating a significant dependence of the peak scores on the presence in the peak sequences of not only highly significant target motifs but also less significant motifs corresponding to the binding sites of the partner transcription factors. A significant correlation was shown between the presence of the target motifs FOXA2 and the partner motifs HNF4G, which found experimental confirmation in the scientific literature, demonstrating the important contribution of the partner transcription factors to the binding of the target transcription factor to DNA and, consequently, their important contribution to the peak score.

## 1. Introduction

All functions during the whole life cycle of living beings are controlled at the genetic level through gene expression. Transcription is the first step in this complex multi-step process, which determines whether a gene is expressed at a given time. The transcription level of a particular gene depends on the cell type, tissue and organ and is regulated depending on the stage of cell differentiation and the stage of organism development [1,2]. Transcription is regulated by a large number of transcriptional regulatory proteins [3,4]. Here, a special role is played by transcription factors (TFs), as they are able to specifically bind to the regulatory regions of genes, determining the composition of multiprotein complexes that form the unique transcription machinery of each gene [5,6]. 

The development of ChIP-seq methods [7,8,9,10] using antibodies for transcription factors has led to the fast-paced accumulation of large bodies of data on the areas where transcription factor binding sites (TFBSs) localize, and thus substantially increased the amount of data on gene regulatory regions. The main advantage of any ChIP-seq method is that it allows the researcher to obtain information on the genome-wide localization of DNA-TF interaction sites in different tissues [11,12] at different stages of organism development [13,14,15] and under different external influences [16] in in vivo settings. For any target TF, the genome-wide ChIP-seq analysis normally reveals tens of thousands of DNA sequences hundreds of nucleotides in length, corresponding to the genomic DNA fragments that have bound to the TF.

Raw data coming from ChIP-seq experiments are stored in data repositories such as GEO [17], ArrayExpress [18], ENA [19] and SRA [20] and are then fed to ChIP-seq analysis pipelines [7,21,22], utilizing peak callers [23], programs that map sequence reads onto a reference genome and identify the regions with the highest coverage on it; these regions being called the “ChIP-seq peaks”. For each ChIP-seq peak, the peak callers make it possible to evaluate the peak score associated with the significance of this peak. The raw data accumulated in ChIP-seq data repositories served as the basis for second-level databases, which contain the results of ChIP-seq experiments classified according to species of organisms, tissue types and cell lines (CODEX [24], BloodChIP [25], hmChIP [26], CistromeDB [27], GTRD [28], ChIP-Atlas [29], TFBSbank [30] and Factorbook [31]), they also generate quality control metrics for the ChIP-seq experiments and have easy-to-use visual interfaces to analyze the localization of ChIP-seq peaks on genomic sequences.

Unfortunately, the peak sequences obtained from a ChIP-seq experiment are much longer than the binding site of any TF, which prevents the accurate localization of the binding site of the target TF and the determination of its size [21,32]. Two classes of computer methods are used to determine the exact locations of potential transcription factor binding sites in ChIP-seq peak sequences. One is based on the identification of ChIP-seq peak sequence regions which are significantly similar to position–weight matrices (PWMs) that describe the binding sites of known TFs and are stored in databases such as HOCOMOCO [33], JASPAR [34] and TRANSFAC [35]. To identify potential TFBSs using position–weight matrices, programs such as HOMER [36], MATCH [37] and MEME Suite [38] are used. Position–weight matrices do not normally consider interpositional dependencies [39,40]. It has been shown that considering dinucleotide dependencies [41], hidden Markov models (HMM) [42,43] and HMM-based TF flexible models (TFFMs) [44] improves the quality of TFBSs recognition in ChIP-seq peak sequences. 

The other approach is based on the de novo detection of significantly overrepresented contextual signals in the sets of the DNA sequences in question. Once the contextual signals have been detected, the degree of their similarity with the position–weight matrices of known TFs stored in the corresponding databases is determined. The use of exhaustive de novo methods that guarantee finding the global optimum in the big data corresponding to the most significant DNA motif is time-consuming and expensive. This explains the researchers’ inclination to consider only a small portion of randomly selected sequences [45] and to use heuristic approaches which can be roughly classified into four types: enumerative [46,47,48,49,50], probabilistic [51,52,53,54,55,56], nature-inspired [57,58] and deep learning [59]. In ChIP-seq peak sequences, such methods can very efficiently identify highly conserved and highly represented contextual signals corresponding to the binding sites of the target TF. At the same time, the efficient identification of more degenerate and poorly represented contextual signals corresponding to the binding sites of partner transcription factors among such data remains an unresolved problem.

Despite the existence of a large number of methods for identifying potential TFBSs, many authors note that the signal of the target factor is not detected in all ChIP-seq peaks [13,60,61,62]. This observation could be due to several factors. (1) TFBSs information stored in the databases is incomplete and the currently available computer-aided methods for identifying TFBSs in ChIP-seq peak sequences do not perform well enough. (2) The physical properties and structural features of DNA, such as melting enthalpy of DNA, DNA bending, groove width, etc., [63,64,65,66,67,68] are ignored. (3) Most computer-aided methods used for the identification of TFBSs are blind to the possibility that more than one variant of the binding site may exist for one transcription factor, which may either be indicative of the presence of more than one conformation of a DNA-protein complex, which, for example, is known for the transcription factors from SREBP family [69], or reflect the different binding preferences of a transcription factor due to post-translational modifications [70,71,72,73]. (4) In some cases, target factors may bind to DNA not directly, but indirectly, through protein–protein interactions with partner TFs and chromatin proteins [62]. 

ChIP-seq peak sequences are characterized by a high-level complexity of contextual organization and may either contain several potential binding sites for target TFs or not contain them at all. Additionally, they may contain quite a few potential binding sites for partner TFs. A wealth of experimental data provide evidence that transcription factors regulate transcription in close cooperation with each other and with other regulatory proteins (transcriptional co-activators and transcriptional co-repressors) [74,75]. This cooperation may result in the formation of, for example, the so-called enhanceosome, when several TFs interacting with a short sequence of a regulatory region form a common surface, which serves as a signal for recruiting coregulatory proteins [76,77]. In the analysis of regulatory regions, this situation corresponds to the presence of stable (frequent) combinations of closely spaced binding sites for transcription factors called the “*cis*-regulatory modules” (CRMs) [78] or composite elements (CEs) [79,80]. Examples of pairs of functionally interacting sites were originally presented in the COMPEL database [79,81]. Interactions between TFs binding to such pairs of sites may give rise to some subtle features of tissue- and stage-specific gene expression [82,83]. It has been shown that transcription factors can bind to the regulatory regions of genes in synergy [84,85] or in competition [86]. Some TFs can facilitate DNA binding to other TFs both by remodeling the nucleosomes and freeing up the regulatory region of the gene for binding to these TFs (as do pioneer TFs, such as FOXA2, Sp1, and PU.1 [87,88,89]), and through direct protein–protein interactions with these TFs [90,91].

Thus, taking into account pairwise and groupwise interactions between transcription factors is necessary for a deeper understanding of gene regulation and for scoring the observed ChIP-seq peaks. Such potentially interdependent TFs are normally sought when using computer-aided methods for the identification of significantly co-occurring contextual motifs corresponding to TFBSs. One group of works addressing this problem relies on the detection of all possible potential TFBSs in the ChIP-seq peak sequences of a particular transcription factor using PWMs from the JASPAR [34], HOCOMOCO [33] and TRANSFAC [35] databases [92,93,94,95]. The other group of works relies on the computer-aided analysis of the whole-genome distribution of ChIP-seq peaks for two TFs from different experiments on the assessment of non-randomness in the intersection of the peaks of these TFs, and the subsequent finding of potential TFBSs in the regions of the peak intersections on the basis of PWMs [96,97,98]. The main limitation of both approaches is the use of a limited set of PWMs for the recognition of partner TFBSs in ChIP-seq peaks; in addition, the second approach requires two independent ChIP-seq experiments under the same cellular conditions. De novo motif discovery methods make it possible to identify all significant contextual signals corresponding to the binding sites in ChIP-seq peak sequences, and target and partner TFs without the need to use PWM databases. 

Despite a large number of methods for identifying TFBSs [36,37,38] and peak callers [23] for detecting ChIP-seq peaks and evaluating their peak scores, no computer-aided methods for estimating the dependence of the peak score of ChIP-seq peaks on the presence of various contextual signals in them are known. 

In this work, we have analyzed the contextual organization of ChIP-seq peak sequences in experiments with 10 TFs belonging to six different superclasses corresponding to the types of their DNA-binding domains [99]. The analysis was carried out using a modified version of our original software package Argo_CUDA version 2.0 [100], with which it is possible to identify sets of significant contextual motifs in the samples of DNA sequences written in a 15-letter IUPAC code [101]. Analysis of the data obtained from each of the ChIP-seq experiments revealed the sets of significant IUPAC motifs (target motifs) corresponded to the binding sites of the target TF studied in each particular experiment and significant IUPAC motifs corresponding to the binding sites of other partner TFs (partner motifs). The motifs recognized in all the ChIP-seq experiments formed the basis of multiple regression models that demonstrated the significant dependence of peak scores of the ChIP-seq peak sequences on the presence of IUPAC motifs in these sequences.

## 2. Results

### 2.1. Preparing Sets of ChIP-Seq Peak Sequences

In this work, we have analyzed the ChIP-seq peak sequences obtained by different research groups [11,12,13,14,15,102,103,104,105,106] in experiments conducted to locate the binding regions for 10 transcription factors (Table 1) in genomic DNA from the CistromeDB database [27]. All ChIP-seq data analyzed in this work were obtained on mouse cells or cell lines. The eighth column of Table 1 contains information about the cell line or type. ChIP-seq experiments were carried out on liver cells [11,102,104,105,106], bone marrow-derived macrophages [12], embryonic stem cells [13,14], erythroid cells from fetal livers [103] and differentiating myoblasts C2C12 [15]. For the purpose of analysis, only experiments with the highest quality scores and containing at least 5000 ChIP-Seq peak sequences were considered. We considered transcription factors that had PWM models presented in the HOCOMOCO database [33] and belong to different TF superclasses according to Wingender’s classification [99]. Only transcription factors from six TF superclasses met these conditions. 

The transcription factors we have chosen regulate the vital functions of cells, including growth, division and differentiation. The transcription factors CEBPA and CEBPB from the C/EBP family are involved in the regulation of the cell cycle and differentiation of various cell types, including blood, liver and adipose tissue cells [107]. The transcription factor NFE2L2/NRF2 is activated in response to inflammation and cell damage, and regulates the expression of antioxidant defense genes [108]. NFE2L2 KO mouse experiments showed that this TF controls the development of the small intestine [109]. SP1 is a ubiquitously expressed transcription factor involved in the control of erythroid cell specification [13]. GATA1 is known to regulate erythropoiesis [110]. FOXA2 is known to be a transcription factor with pioneering functions and is involved in the regulation of morphogenesis [111]. FOXO1, known to be a regulator of the cell cycle, apoptosis and oxidative stress, is involved in the regulation of placental and cardiovascular morphogenesis [112,113]. NFYA controls the expression of multiple genes involved in cell cycle regulation and steps down as a regulator of the stemness and proliferation of mouse embryonic stem cells (mESCs) and human hematopoietic stem cells (hHSCs) [114]. MEF2D is involved in the differentiation of muscle cells [15]. STAT5B is involved in the regulation of the differentiation of osteoblasts, adipocytes and neuronal cells [115,116,117].

Based on the results of each ChIP-seq experiment, DNA sequences were obtained in the [−100;+100] region relative to the maxima of the ChIP-seq peaks. Next, the ChIP-seq peaks were ranked according to the peak scores (PSs), which reflect the enrichment of the peaks. Larger numbers of peak scores represent more confident peak calls. 

To ensure that the comparative characteristics of IUPAC motifs recognized do not depend on the size of the training sets, we selected 5000 peak sequences considering the maximum peak scores for each of the 10 ChIP-seq experiments. From these sequences, training sets were compiled. The remaining sequences were included in the control sets for each ChIP-seq experiment. DNA sequences corresponding to ChIP-seq peaks were extracted from the GRCm38_97 mouse genomic assembly available in the EMBL database according to CistromeDB annotation [118].

As can be seen from Table 1, the numbers of entries in the control sets of ChIP-seq peak sequences ranged from 975 for NFYA to 29,789 for MEF2D. 

### 2.2. Identification of Significant Oligonucleotide Motifs in the ChIP-Seq Sequences

In the training data sets created for each of the ChIP-seq experiments and consisting of 5000 DNA sequences located in the [−100;+100] regions relative to the ChIP-seq peaks, we de novo identified sets of significant degenerate motifs written in the 15-letter IUPAC code (A,T,G,C, R = G/A, Y = T/C, M = A/C, K = G/T, W = A /T, S = G/C, B = T/G/C, V = A/G/C, H = A/T/C, D = A/T/G, N = A/T/G/C). The identification of significant IUPAC motifs was carried out using our original de novo motif discovery system Argo_CUDA [100] with some improvements (see Section 4). Unlike heuristic methods, the de novo motif discovery GPU-based Argo_CUDA assesses the significance of all possible IUPAC motifs of a given length, which guarantees that a global optimum will be found. At the same time, IUPAC motifs were considered to be significant if they were significantly overrepresented in the set of sequences being analyzed compared to the values of abundance expected to have been observed for random reasons. A detailed description of the significance criterion (1) and the boundary values used are also provided in the Section 4.

As a result of the analysis, sets of significant IUPAC motifs were identified in each of the ten training sets corresponding to ChIP-seq experiments with different transcription factors (see Table 2). 

Table 2 shows that although the number of DNA sequences in the sets of the results of different ChIP-seq experiments was the same (N = 5000), and the same boundary values were used for the significance criterion (1), the number of motifs identified in each ChIP-seq experiment and their maximum significance vary strongly. Thus, there was an ~1.8-fold variation in the number of all motifs identified (at *P_Bonf_* (*n*,*N*) < *p*_0_ = 10^−2^) in these ChIP-seq experiments. The largest number of significant motifs (270) was found for NFYA, and the least one (151) was found for CEBPA. An average of 206 IUPAC motifs were identified in all 10 ChIP-seq data sets. The highest maximum significance of the motifs was observed in the ChIP-seq experiment with FOXA2 (*P_Bonf_* (*n*,*N*) = 10^−1462^), and the lowest, with FOXO1 (*P_Bonf_* (*n*,*N*) = 10^−447^). As can be noted, fewer significant motifs were in the training set **FOXA2_L_** (202) than in the training set **FOXO1_L_** (220). On the whole, there was a negative correlation (r = −0.39) between the number of motifs detected and their maximum significance. For each of the sets, we inferred the number of the most significant (*P_Bonf_* (*n*,*N*) < *p*_0_ = 10^−30^) motifs detected and its ~2.6-fold variation. The maximal number of the most significant motifs (81) was found for NFYA, and the minimum number (31) was found for for GATA1. An average of 54 most-significant IUPAC motifs were detected in all 10 ChIP-seq datasets. As can be noted, the number of the most significant motifs detected in each ChIP-seq experiment strongly correlates with the total number of all motifs detected in it (r = 0.95) and negatively correlates with the maximum significance of the motifs detected (r = −0.31). 

**Figure 1:**
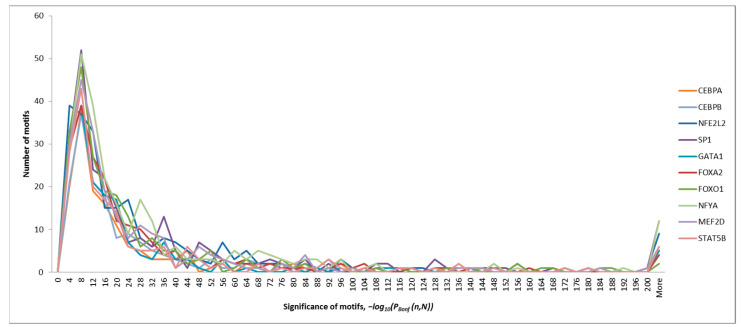
Dependence of the number of IUPAC motifs (*y*-axis) detected in ChIP-seq experiments with 10 transcription factors on the significance of these motifs (*x*-axis).

The dependence of the number of IUPAC motifs detected in each of the sequence sets on the motif significance is shown in Figure 1. 

As can be noted, all the sets of motifs we obtained had a similar distribution pattern: the region corresponding to the probability of observing them for random reasons *P_Bonf_* (*n*,*N*) ϵ [10^−30^; 10^−2^] has a pronounced peak in the number of the motifs detected, which further turns into a very long tail. 

Table 3 shows examples and characteristics of the most significant (*P_Bonf_* (*n*,*N*) < 10^−30^) IUPAC motifs detected in the training set **FOXA2_L_** for ChIP-seq peak sequences in the FOXA2 experiment (ID 3266 in CistromeDB). FOXA2, known as hepatocyte nuclear factor 3-beta (HNF-3B), belongs to the FOXA subfamily. This subfamily, in turn, belongs to the FOX family, whose proteins contain a relatively conserved DNA binding domain known as the winged-helix or forkhead domain.

For example, the most significant motif TRTWKACH = (T)(A/G)(T)(A/T)(G/T)(A)(C)(A/T/C) detected in **FOXA2_L_** was present in n = 3345 out of N = 5000 sequences (F ~ 0.67), the expectation to observe it for random reasons was 764 sequences (Q ~ 0.15), and the Bonferroni-corrected binomial probability of observing it in at least 3345 out of 5000 sequences for random reasons was *P_Bonf_* (3345, 5000) = 10^−1462.3^.

### 2.3. Multiple Regression Model-Based Assessment of the Dependence of ChIP-Seq Peak Scores on the Presence of Significant IUPAC Motifs in Them

To assess the relationship between the presence of motifs in a ChIP-seq peak sequence and the natural logarithm of their peak scores (*ln(PS)*), multiple regression models were built (see Section 4) on the training sets for each ChIP-seq experiment using STATISTICA (StatSoft^TM^, Tulsa, OK, USA). The regression coefficients obtained were then used to assess the correlation between the peak scores predicted by the multiple regression models and the peak scores that were actually observed in the training and control sets for each ChIP-seq experiment. The results of the analysis and the correlation coefficients obtained for the training and control sets are shown in Table 4.

#### 2.3.1. Assessment of the Dependence of Peak Scores on the Presence of Significant IUPAC Motifs in ChIP-Seq Peaks in the Experiment with the Transcription Factor FOXA2

Let us consider in detail the construction of a multiple regression model, using the analysis of ChIP-seq data from the FOXA2 experiment as an example. For each of the 5000 DNA sequences of the ChIP-seq peaks in the [−100;100] region relative to its maximum value in the training set **FOXA2_L_**, the abundance of each of the 202 motifs previously detected in this IUPAC motif set was assessed. The abundance of a motif in a sequence means the number of observations of the given motif along the entire length of the DNA sequence in both of its strands. The abundance vectors, obtained in this way for all motifs considered, served as independent variables. The dependent variable was information about the natural logarithm of the peak scores of the sequences in the training set. To assess the dependence of peak scores on the abundance of IUPAC motifs in these ChIP-seq peaks, a multiple regression model was built using STATISTICA (StatSoft^TM^, Tulsa, OK, USA). Table 5 shows examples of independent variables with significant (*p* < 0.05) regression coefficients and their characteristics. A complete table of the regression coefficients for all IUPAC motifs considered is provided in “Supplementary Materials”, Table S1. 

Of the total number of independent variables considered that corresponded to the abundance of 202 significant IUPAC motifs, only 30 (15%) had significant (*p* < 0.05) regression coefficients. 

**Figure 2:**
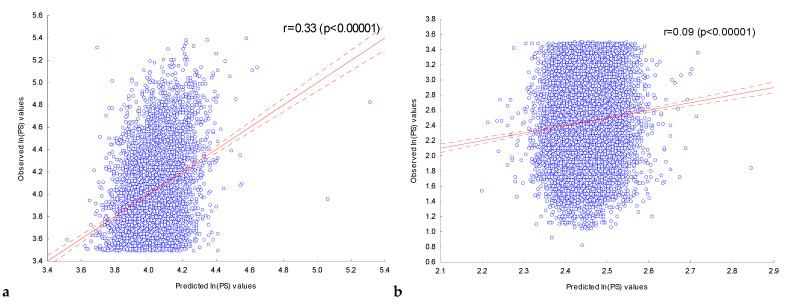
Dependence of the observed value of *ln(PS)_Obs_* for ChIP-seq peak sequences in the training (**a**) and control (**b**) FOXA2 sets on the expected value. The expected value of *ln(PS)_Exp_* for the peaks was predicted using the multiple regression model on the presence of 202 significant IUPAC motifs previously detected in the training set **FOXA2_L_**. Solid and dashed lines represent the regression line and the bounds of its 95% confidence interval as calculated using STATISTICA (StatSoft^TM^, Tulsa, OK, USA). r is the linear correlation coefficient and *p* is its statistical significance.

Figure 2a shows the dependence of the observed value of *ln(PS)_Obs_* for the ChIP-seq peak sequences in the training set **FOXA2_L_** on the expected value of their *ln(PS)_Exp_* calculated with the multiple regression model using all 202 independent variables. The observed *ln(PS)_Obs_* and expected *ln(PS)_Exp_* values were significantly (*p* < 10^−5^) correlated (r = 0.33). 

This regression model was used to predict the expected *ln(PS)_Exp_* values for ChIP-seq peak sequences in the control set **FOXA2_C_**. Figure 2b shows the plotted relationship between the observed *ln(PS)_Obs_* and the expected *ln(PS)_Exp_* values predicted. For these values, a significant (*p* < 10^−5^) correlation was also observed (r = 0.09).

Thus, the information about the presence of ChIP-seq peaks in the DNA sequences in the FOXA2 experiment allows conclusions to be drawn about the peak score values for the ChIP-seq peaks of FOXA2, which had not previously been used for detecting significant IUPAC motifs or for constructing multiple regression models. 

#### 2.3.2. Assessment of the Dependence of Peak Scores in Experiments with Nine Transcription Factors on the Presence of Significant IUPAC Motifs in Their ChIP-Seq Peaks

Similarly, multiple regression models were built on the training sets of the remaining nine transcription factors. These models were used to predict the peak score of ChIP-seq peak sequences in the training and control sets (Table 4, “Supplementary Materials”, Figure S1). The analysis performed showed that the correlation coefficients r in predicting the observed values of peak scores were significant for all ten training sets. They ranged from r = 0.3 for the **GATA1_L_** set to r = 0.57 for the **SP1_L_** set. On average, the training sets were characterized by r = 0.38. The r value for each of the training sets correlated non-significantly (*p* > 0.05) with the number of previously detected significant IUPAC motifs in it (r = 0.58). 

The vast majority (8 out of 10) of the control sets had significant correlation coefficients in predicting the observed values of peak scores. At the same time, it was not possible to build a reliable prediction model for the **NFE2L2_C_** and **GATA1_C_** sets. On average, significant correlation coefficients were r = 0.09 and ranged from r = 0.06 for the **SP1_C_** set to r = 0.13 for the **CEBPB_C_** set. A comparison of the correlation coefficients r for the training and control sets shows that the values were significantly lower for the latter. The analysis performed did not reveal significant (*p* < 0.05) dependencies of the r values for the control sets on the size of these sets or on the r values for the corresponding training sets or on the maximum peak score in the sets. 

Thus, it can be hypothesized that the DNA sequences in the ChIP-seq peaks with the highest peak scores are consistent with a model of context organization which is different to that for ChIP-seq peaks with low peak scores. 

## 3. Discussion

### 3.1. Assessment of the Contribution of the Most Significant IUPAC Motifs to the Prediction of the Peak Scores

As noted previously in the analysis of Figure 1, all of the significant IUPAC motifs detected in each training set were divided into two groups: (1) the largest group, consisting of IUPAC motifs whose probability of being observed was *P_Bonf_* (*n*,*N*) ϵ [10^−30^; 10^−2^] and (2) the most significant motifs (*P_Bonf_* (*n*,*N*) < 10^−30^) in the tail of the distribution. It can be assumed that taking the presence of only the most significant IUPAC motifs in the DNA sequence of a ChIP-seq peak into account will be sufficient to predict its peak score. In order to assess how strongly the peak score of a ChIP-seq peak sequence depends on the presence of only the most significant IUPAC motifs in it, we built a multiple regression model for predicting the peak score based on IUPAC motifs only from the group with *P_Bonf_* (*n*,*N*) < 10^−30^. The model was built for the sets with ChIP-seq peak sequences obtained in the experiments with FOXA2 and SP1. 

#### 3.1.1. Assessment of the Dependence of the Peak Scores in the Experiment with the Transcription Factor FOXA2 on the Presence of Only the Most Significant IUPAC Motifs in the Peaks

A multiple regression model was built for the **FOXA2_L_** training set with STATISTICA (StatSoft^TM^, Tulsa, OK, USA) using only the 50 most significant (*P_Bonf_* (*n*,*N*) < 10^−30^) IUPAC motifs detected in it (the motifs are listed in the “Supplementary Data”). Table 6 shows examples of independent variables with significant (*p* < 0.05) regression coefficients and their characteristics. A complete table of the regression coefficients for all IUPAC motifs considered is provided in the Supplementary Materials, Table S2.

As can be seen from Table 6, of all the independent variables corresponding to the abundance of the 50 most significant IUPAC motifs, only 11 (22%) had significant (*p* < 0.05) regression coefficients. 

**Figure 3:**
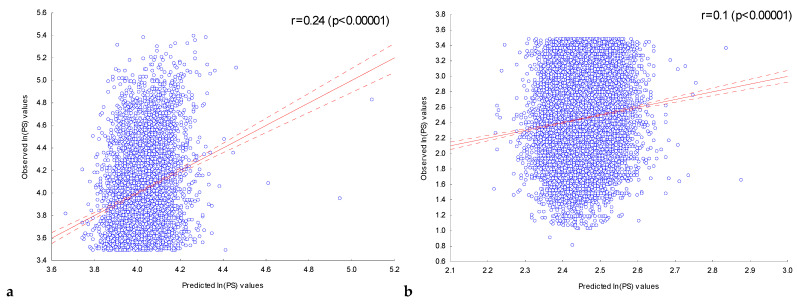
Dependence of the observed values of *ln(PS)_Obs_* for ChIP-seq peak sequences from (**a**) the training set **FOXA2_L_** and (**b**) the control set **FOXA2_C_** on their expected values. The expected value of *ln(PS)_Exp_* for the ChIP-seq peaks was predicted using a multiple regression model for the presence of the most significant (*P_Bonf_* (*n*,*N*) < 10^−30^) IUPAC motifs previously detected in the training set **FOXA2_L_**. Solid and dashed lines represent the regression line and the bounds of its 95% confidence interval as calculated using STATISTICA (StatSoft^TM^, Tulsa, OK, USA). r is the linear correlation coefficient and *p* is its statistical significance.

With the multiple regression model constructed, peak scores were predicted for the sequences in the training set **FOXA2_L_** and the control set **FOXA2_C_**. Figure 3 shows the dependencies of the observed *ln(PS)_Obs_* values for the ChIP-seq peak sequences of the training set **FOXA2_L_** (Figure 3a) and the control set **FOXA2_C_** (Figure 3b) on their expected *ln(PS)_Exp_* values calculated with the multiple regression model constructed, using all 50 independent variables. 

For both sets, the observed *ln(PS)_Obs_* and expected *ln(PS)_Exp_* values were significantly (*p* < 10^−5^) correlated. At the same time, for the training set **FOXA2_L_**, the use of only the most significant (*P_Bonf_* (*n*,*N*) < 10^−30^) IUPAC motifs led to a lower correlation (r = 0.24) than the use of all 202 significant motifs (*P_Bonf_* (*n*,*N*) < 10^−2^), r = 0.33. On the other hand, taking into account the localization of only the most significant (*P_Bonf_* (*n*,*N*) < 10^−30^) IUPAC motifs for the control set **FOXA2_C_** allowed us to obtain a slightly higher correlation (r = 0.1) than was obtained using all 202 significant IUPAC motifs (r = 0.09). On the whole, we can conclude that using information about localization in the ChIP-seq sequences of only the most significant motifs without taking into account other motifs does not allow predicting the peak score values with the greatest accuracy in all cases. 

#### 3.1.2. Assessment of the Dependence of Peak Scores for Peak Sequences in the Experiment with the Transcription Factor SP1 on the Presence of Only the Most Significant IUPAC Motifs in Them

A multiple regression model was built for the training set **SP1_L_** with STATISTICA (StatSoft^TM^, Tulsa, OK, USA) using only the 80 most significant (*P_Bonf_* (*n*,*N*) < 10^−30^) IUPAC motifs detected in it (the motifs are listed in the “Supplementary Data”). Table 7 shows examples of independent variables with significant (*p* < 0.05) regression coefficients and their characteristics. A complete table of the regression coefficients for all IUPAC motifs considered is provided in the Supplementary Materials, Table S3.

As can be seen from Table 7, of the 80 independent variables considered, 29 had significant (*p* < 0.05) regression coefficients (36%). 

**Figure 4:**
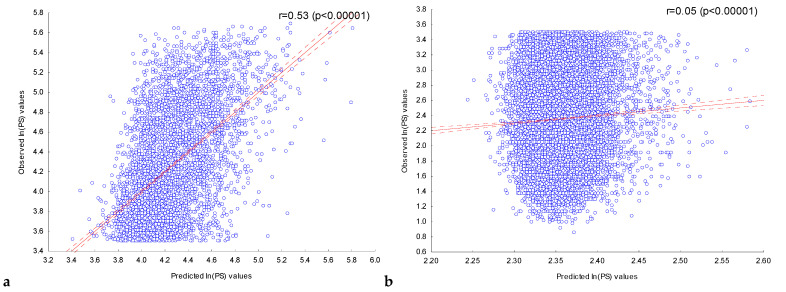
Dependence of the observed values of ln(PS)_Obs_ for ChIP-seq peak sequences from (**a**) the training set **SP1_L_** and (**b**) the control set **SP1_C_** on their expected values. The expected value of ln(PS)_Exp_ for the ChIP-seq peaks was predicted using a multiple regression model for the presence of the most significant (*P_Bonf_* (*n*,*N*) < 10^−30^) IUPAC motifs previously detected in the training set **SP1_L_**. Solid and dashed lines represent the regression line and the bounds of its 95% confidence interval as calculated using STATISTICA (StatSoft^TM^, Tulsa, OK, USA). r is the linear correlation coefficient and *p* is its statistical significance.

With the multiple regression model constructed, peak scores were predicted for the sequences of the training set **SP1_L_** and the control set **SP1_C_**. Figure 4 shows the dependencies of the observed *ln(PS)_Obs_* values for the ChIP-seq peak sequences of the training set **SP1_L_** (Figure 4a) and the control set **SP1_C_** (Figure 4b) on their expected *ln(PS)_Exp_* value calculated with the multiple regression model constructed, using all independent variables corresponding to the abundances of the 80 most significant IUPAC motifs. 

For both sets, the observed *ln(PS)_Obs_* and expected *ln(PS)_Exp_* values were significantly (*p* < 10^−5^) correlated. In the case of the training set **SP1_L_**, the use of only the 80 most significant (*P_Bonf_* (*n*,*N*) < 10^−30^) IUPAC motifs led to a somewhat lower correlation (r = 0.53) than the use of all 243 significant IUPAC motifs (*P_Bonf_* (*n*,*N*) < 10^−2^), r = 0.57 (Figure S1, Supplementary Materials, Table 4). In the case of the control set **SP1_C_**, the use of information about the localization of only the 80 most significant (*P_Bonf_* (*n*,*N*) < 10^−30^) IUPAC motifs also led to a somewhat lower correlation (r = 0.05) than the use of all 243 significant IUPAC motifs (*P_Bonf_* (*n*,*N*) < 10^−2^), r = 0.06 (Figure S1, Supplementary Materials, Table 4). 

Thus, the analysis of data on the ChIP-seq experiments with two TFs allows us to conclude that, to predict the peak scores of ChIP-seq peak sequences, it is necessary to use information about the localization of both the most significant motifs and other IUPAC motifs in them. 

### 3.2. Assessment of the Correlations between the Presence of the Target IUPAC Motifs and Partner IUPAC Motifs in the Peak Sequences in the Experiment with Transcription Factor FOXA2

While we were building multiple regression models for all of the transcription factors considered, the analysis of the regression coefficients (Table 5, Table 6 and Table 7) suggested that only a small fraction of them were significant (*p* < 0.05). One of the reasons for this may be the internal interdependencies of the presence of different IUPAC motifs in the ChIP-seq peak sequences. 

#### 3.2.1. Assessment of the Correlations between the Co-Occurrences of the Most Significant (*P_Bonf_* (*n*,*N*) < 10^−30^) IUPAC Motifs in the Peak Sequences

**Figure 5:**
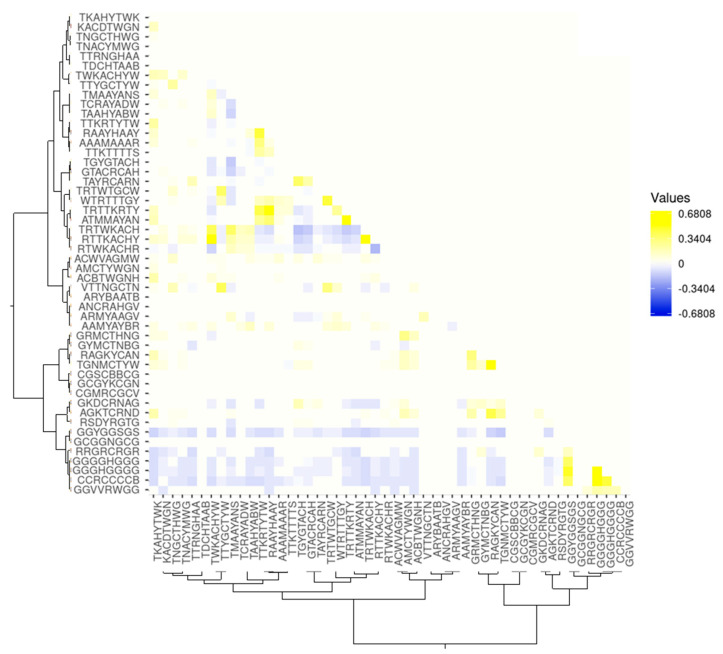
Heat map of significant (*p* < 0.05) pairwise correlations between the co-occurrence of the 50 most significant (*p* < 10^−30^) IUPAC motifs found in [−100;+100] DNA sequences relative to the ChIP-seq FOXA2 peak maximum. Blue, a negative correlation; yellow, a positive correlation; white, a neutral situation when the correlation between the co-occurring IUPAC motifs failed to reach significance. Clustering was carried out according to the degree of their similarity using the Kullback–Leibler distance in the web system STAMP [122].

To test the proposed assumption, we assessed the correlations between the co-occurrences of the most significant (*p* < 10^−30^) motifs in the ChIP-seq peak sequences from the **FOXA2_L_** set using the phi-coefficient [120] to assess paired correlations. Each IUPAC motif from the pair of motifs considered was assumed to be located in the ChIP-seq peak sequence if it had been found at least once in either of the DNA strands. Figure 5 shows a heat map of the pairwise correlations obtained (built with the Heatmapper tool [121] (see Section 4)). 

Figure 5 shows that the motifs have very highly significant (*p* < 0.05) correlations between the co-occurrences of IUPAC motifs in the ChIP-seq peak sequences. Moreover, both significant positive and negative correlations are observed for their co-localization with each other. Clustering according to the degree of similarity in the IUPAC motifs using Kullback–Leibler distance in the web system STAMP [120] showed that all the motifs fall into two large classes. Annotation (see Section 4) of IUPAC motifs using the web resource Tomtom [123] showed that the vast majority of the motifs in Group 1 had a significant similarity with known PWMs for the binding sites of the transcription factor FOXA2. The vast majority of the motifs in Group 2 had a significant similarity with the PWMs of the binding sites for partner transcription factors. As can be seen from the heat map, Group 2 motifs have a subgroup of significantly often co-occurring motifs, but the correlations of their co-occurrence with the motifs of the FOXA2 binding sites are negative. Tomtom annotation showed that these motifs may significantly (*p* < 0.001) correspond to the binding sites of the transcription factor SP1. 

Analysis of pairwise correlations of the co-localization of IUPAC motifs from Group 1 demonstrates a substantial prevalence of positive correlations. At the same time, a small number of negative correlations are also observed. 

All this points to complex relationships between TFs in the regulatory regions of genes and a high heterogeneity of data being analyzed. In addition, it was shown that the IUPAC motifs (partner motifs) corresponding to the binding sites of the partner TFs can both positively and negatively correlate with IUPAC motifs (target motifs) corresponding to the binding sites of the target TF FOXA2. 

#### 3.2.2. Assessment of the Dependence of the Peak Scores of Peak Sequences on the Presence of Only the Most Significant (*P_Bonf_* (*n*,*N*) < 10^−30^) Target Motifs in Them 

To assess the contribution of the target motifs to the efficiency of peak score prediction, we built a multiple regression model with the use of only the most significant (*P_Bonf_* (*n*,*N*) < 10^−30^) target motifs corresponding to the binding sites of the target transcription factor FOXA2. To this end, the annotation of the most significant (*p* < 10^−30^) IUPAC motifs obtained from analysis of ChIP-seq peak sequences in the experiment with FOXA2 was carried out using the web system Tomtom [123] (Section 4), and the motifs that had a significant similarity (*p* < 0.001) with the FOXA2 binding sites PWM were identified. As was found, out of the 50 IUPAC motifs considered, only 22 (44%) were the target motifs. Information about the localization of these motifs in the ChIP-seq peak sequences of the training set **FOXA2_L_** was used to build a multiple regression model predicting the peak scores of these sequences. Table 8 shows examples of independent variables with significant (*p* < 0.05) regression coefficients and their characteristics. A complete table of the regression coefficients for all IUPAC motifs considered is provided in the Supplementary Materials, Table S4.

Table 8 shows that nine (41%) of the twenty-two independent variables considered had significant (*p* < 0.05) regression coefficients. 

**Figure 6:**
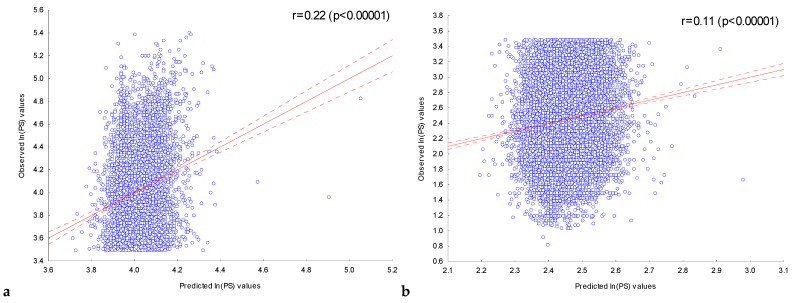
Dependence of the observed values of *ln(PS)_Obs_* for ChIP-seq peak sequences from (**a**) the training set **FOXA2_L_** and (**b**) the control set **FOXA2_C_** on their expected values. The expected value of *ln(PS)_Exp_* for ChIP-seq peaks was predicted using a multiple regression model for the presence of the most significant (*P_Bonf_* (*n*,*N*) < 10^−30^) target motifs previously detected in the training set **FOXA2_L_**. Solid and dashed lines represent the regression line and the bounds of its 95% confidence interval as calculated using STATISTICA (StatSoft^TM^, Tulsa, OK, USA). r is the linear correlation coefficient and *p* is its statistical significance.

This multiple regression model was used to predict the peak scores of the sequences for the training set **FOXA2_L_** and the control set **FOXA2_C_**. Figure 6 shows the observed *ln(PS)_Obs_* value for the ChIP-seq peak sequences of the training set **FOXA2_L_** (Figure 6a) and the control set **FOXA2_C_** (Figure 6b) versus their expected *ln(PS)_Exp_* values calculated with the multiple regression model using information about the localization of the 22 most significant (*P_Bonf_* (*n*,*N*) < 10^−30^) target motifs. 

For both sets, the observed *ln(PS)_Obs_* and expected *ln(PS)_Exp_* values were significantly (*p* < 10^−5^) correlated. In the case of the training set **FOXA2_L_**, the use of only the 22 most significant (*P_Bonf_* (*n*,*N*) < 10^−30^) target motifs led to a slightly lower correlation (r= 0.22) than the use of all 50 of the most significant IUPAC motifs, r = 0.24 (Figure 3a). In the case of the control set **FOXA2_C_**, the use of information about the localization of only the 22 most significant (*P_Bonf_* (*n*,*N*) < 10^−30^) target motifs led to a slightly increased correlation coefficient (r = 0.11) compared to that obtained from using all 50 of the most significant motifs (r = 0.1) (Figure 3b). 

Thus, it can be concluded that the use of complete information about the localization of peaks in ChIP-seq sequences of all previously identified significant IUPAC motifs is most effective in predicting the peak scores of ChIP-seq peak sequences in the training sets. Only using information about the presence of the most significant target motifs makes it possible to slightly improve the quality of peak score prediction for ChIP-seq peak sequences in the control sets. 

#### 3.2.3. Analysis of the Correlations between the Presence of Target Motifs and the Motifs of the Partner Transcription Factors in the ChIP-Seq Peak Sequences

As can be seen from the analysis of the results obtained, the target motifs make a significant contribution to the prediction of ChIP-seq peak scores; however, sole reliance on target motifs leads to a decrease in prediction quality for ChIP-seq peak sequences that have the highest peak scores and are included in the training sets. To identify transcription factors that can be partners to the target transcription factor FOXA2 in this set and contribute to its functioning when it interacts with the ChIP-seq peak sequences in **FOXA2_L_**, we performed a functional annotation of 28 most significant (*P_Bonf_* (*n*,*N*) < 10^−30^) IUPAC motifs previously detected in this set and not targeted. The annotation was carried out using the web system Tomtom [123] as described in the Section 4. The results of the annotation are shown in Table 9. 

We assessed how the motifs corresponding to the binding sites of the partner TF and the motifs corresponding to the binding sites of the target TF FOXA2 co-occur in the **FOXA2_L_** ChIP-seq peak sequences. Here, in our opinion, the ChIP-seq peak sequence contains the binding site for the TF considered if at least one of the motifs that are significantly similar to the position–weight matrix of this TF is located in this sequence. Consequently, the ChIP-seq peak sequence does not contain a TF binding site if none of the motifs which are significantly similar to the position–weight matrix of this TF are located in the sequence. To exclude from consideration the correlations accounted for by the heterogeneity of the **FOXA2_L_** set and the peculiarities of its nucleotide context, we assessed the significance of the IUPAC motifs detected and the correlations calculated on their basis using the shuffling procedure (see the Section 4). Table 10 shows the correlation coefficients obtained using the phi-coefficient between the motifs of the partner TF and the motifs of the target TF for the given FOXA2 set. 

As was found, only two partner transcription factors, HNF4G and MAFG, were characterized by a significant (*p* < 10^−5^) correlation of the co-occurrence of their potential binding sites with the potential binding sites for FOXA2. Thus, these TFs can potentially interact with FOXA2 in the regions of ChIP-seq peaks and function synergistically with it. 

#### 3.2.4. Experimental Data Confirming the Functional Relevance of the Associations Revealed between the Target and Partner Motifs

We wanted to assess the extent to which the information on the co-occurrence of potential binding sites for partner TFs obtained using our proposed approaches is consistent with the experimental data obtained previously and described in the literature. To this end, we analyzed scientific publications describing experimental studies of synergistic interactions with FOXA2. The analysis revealed a number of reports providing experimental evidence for the significant associations that we have found between the target motifs of FOXA2 and the partner motifs of HNF4G in the ChIP-seq peak sequences (Table 11). 

In particular, there is evidence of molecular interactions between FOXA2 and factors from the HNF4 subfamily at human and mouse regulatory DNA elements: (1) co-transfection experiments with the expression vectors for HNF3beta and HNF4 revealed that these factors may bind to the enhancer of the human gene APOB and act synergistically to enhance the intestinal expression of APOB [124]; (2) analyzing DNA regions from ChIP-seq experiments both for FOXA2 and for HNF4a, Wallerman co-workers found that almost half of the FOXA2 peaks were co-localizing with HNF4A peaks, often at a very close distance and with both motifs present [125]; (3) using RNA-seq and ChIP-seq libraries generated from embryonic hepatoblasts and adult mice liver, Alder co-workers showed that the key hepatic TFs HNF4A and FOXA2 occupy enhancers and control target gene expression in a differentiation-dependent manner [126]; (4) in addition, Ceelie co-workers showed that the co-binding of FOXA2 and HNF4A, as well as SP1, with the human prothrombin (F2) enhancer is necessary to ensure an appropriate level of prothrombin expression [127]; (5) analyzing DNA regions from ChIP-seq experiments for FOXA2 and HNF4A in the adult mouse liver, Hoffman co-workers demonstrated FOXA2 and HNF4A collaborations in maintaining the expression of genes that were initially co-expressed in the developing pancreas and liver. They identified 3236 loci in the liver that were co-bound by FOXA2 and HNF4A [128]. 

Unfortunately, we have not yet found scientific publications that experimentally confirm the synergistic interactions between MAFG and FOXA2. Thus, MAFG may be used as a target for the experimental study of its possible synergistic interactions with FOXA2. 

## 4. Methods and Materials

### 4.1. Brief Description of the De Novo Motif Discovery System Argo_CUDA

#### 4.1.1. Description of the Criterion used for the Significance of IUPAC Motifs Detected by Argo_CUDA 

The purpose of Argo_CUDA is to iteratively identify significant IUPAC motifs of a fixed length *k* = 8 bp in a **Pos** set consisting of *N* sequences of interest of length *L*. A specific IUPAC motif is considered significant if it is overrepresented in the given set of sequences in **Pos**, its expected presence in **Pos** for random reasons is low, and the probability of its observation in **Pos** for random reasons is significantly low; that is, condition (1) is satisfied.

● *F* > *f*_0_

● *Q* < *q*_0_ (1)

● *P_Bonf_* (*n*,*N*) < *p*_0_

Here *F* = *n/N* is the abundance of the motif in **Pos**; that is, the proportion of **Pos** sequences in which the motif occurs at least once; *n* is the number of **Pos** sequences in which the motif occurs at least once; *Q* is the expected abundance of the motif in **Pos**; that is, the proportion of **Pos** sequences in which the motif is expected to appear at least once for random reasons; the binomial probability *P_Bonf_(n,N)* of observing a motif in at least *n* of *N* sequences of the given set **Pos** for random reasons. *P_Bonf_(n,N)* is calculated taking into account the Bonferroni correction (see Supplementary Materials). *f*_0_, *q*_0_ and *p*_0_ are user-selectable limit values. 

#### 4.1.2. Brief Description of the Argo_CUDA Algorithm

**Figure 7:**
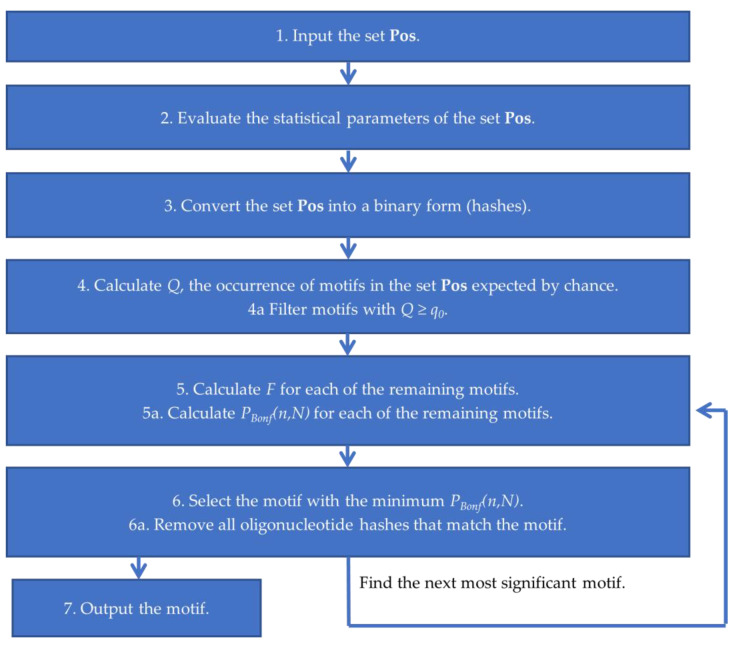
Block diagram for Argo_CUDA, a motif discovery algorithm.

Figure 7 shows a block diagram for our proposed algorithm. This de novo motif discovery algorithm is exhaustive and estimates the significance of the abundance of all 15*^k^* possible IUPAC motifs of fixed length *k* in the given set **Pos**. Unlike the enumeration and probabilistic approaches, Argo_CUDA can reliably find the global optimum and identify the most significant motif. 

In step (1), the given set **Pos** containing the nucleotide sequences of ChIP-seq peaks is fed to Argo_CUDA. In step (2), the frequency characteristics of the set **Pos** are estimated taking into account the Markov level (up to the 2nd order). At the request of the user, the Bernoulli model with a Markov level of 0 and equal frequencies of nucleotides can be used (P_A_ = P_T_ = P_G_ = P_C_ = 0.25). In step (3), Argo_CUDA converts the **Pos** sequences into an array of hashes corresponding to oligonucleotides of length 8 (Table 12). For example, the oligonucleotide **atataaaa** can be represented as a 4-byte binary hash 0001 0010 0001 0010 0001 0001 0001 0001. Thus, each nucleotide sequence of length *L* is converted into an array of 4-byte hashes of length *L* − *k* + 1, where *k* is the length of the oligonucleotide motif. 

**Figure 8:**
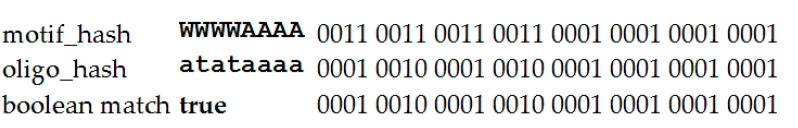
An example of comparing the motif **WWWWAAAA** and the oligonucleotide **atataaaa**. Even if any single position of the motif does not match the oligonucleotide, the result of the bitwise “AND” operation is zero.

In step (**4**), for each of the possible 15^k^ IUPAC motifs, an estimate is made of its expected occurrence *Q* in **Pos** for random reasons (see Supplementary Materials). It should be noted that a large proportion of all possible motifs may be irrelevant from a biological point of view due to their excessive degeneration (for example, **NNNNNNNN**). There is no need to waste program execution time in assessing the abundance of such motifs in the **Pos** set of DNA sequences, and so such motifs can be eliminated immediately and not considered further. Thus, if for the given motif *Q > q*_0_, then according to criterion (1), the motif is excluded from consideration, since it occurs by chance too often. This step significantly narrows the number of possible motifs and substantially speeds up the program. Figure S2 (Supplementary Materials) shows that, for example, at *q*_0_ = 30% in the set of randomly generated sequences, the number of motifs being considered decreases from 15^8^ ~ 2.6 × 10^9^ to ~8.6 × 10^8^, which amounts to about a three-fold decrease. In stage (5), the abundance *F* = *n/N* and statistical significance *P_Bonf_(n,N)* are calculated for the motifs remaining after filtering. Here, the estimation, n, of the number of **Pos** sequences containing the motif of interest is made using the GPU. In order to substantially accelerate the comparison of the correspondence between DNA regions and IUPAC motifs, we used binary representations of both motifs and oligonucleotides of DNA sets (Figure 8). A comparison of a motif of length 8 with a DNA region of the same length is performed in one bitwise “AND” operation and one comparison operation instead of eight operations for assessing the correspondence between a nucleotide and a letter of the IUPAC code, which significantly reduces the program runtime: *boolean match* = *(oligo_hash & motif_hash)* = *= oligo_hash*. 

The proposed optimization methods can substantially speed up the algorithm; however, it will still require a huge amount of computing power. That is why we used high-performance graphics accelerators (GPUs) to explore motif abundance. GPUs are especially powerful for deep parallelization tasks, especially when the data are independent. In step (**6**), the most significant motif with the smallest *P_Bonf_(n,N)* that satisfies the significance criterion (1) is selected. This motif is retained, and all the oligonucleotide hashes that correspond to it are removed from **Pos**. Steps 5 and 6 are repeated to identify the next most significant IUPAC motif. After it became impossible to identify any IUPAC motif that satisfies the significance criterion (1) in **Pos**, all previously detected significant motifs at stage (7) are saved to a file and Argo_CUDA stops. 

#### 4.1.3. Parameter Values Used to Identify Significant IUPAC Motifs in ChIP-Seq Peak Sequences

Our original development Argo_CUDA was used to identify conserved motifs in the sets of peak sequences obtained from ChIP-seq experiments with ten transcription factors (Table 1). Any motif detected was considered to meet the significance criterion (1) if:

(1) It was located in at least *f*_0_ = 1% of the ChIP-seq peak sequences;
(2) Its expected abundance for random reasons was not more than *q*_0_ = 30%. The expected abundance was calculated taking into account the 3rd order Markov level.
(3) The Bonferroni-corrected binomial probability of observing the motif for random reasons was not more than *p*_0_ = 0.01.

Both DNA strands were examined.

### 4.2. Building a Multiple Regression Model

The general purpose of a multiple regression approach is to analyze the relationship between several independent variables (also called predictors) and a dependent variable. The objective of a multiple regression analysis is to use independent variables to predict the value of a single dependent variable. The value of each predictor is weighted, and the resulting weights reflect the contribution of each predictor to the overall prediction.

The multiple linear regression equation is

*Y* = *B*_0_ + *B*_1_*X*_1_ + *B*_2_*X*_2_ + ⋯+ *B_n_X_n_ + e.*

Here, *Y* is the dependent variable, *X_1_,…,X_n_* are n independent variables, *B_0_* is the *Y*-intercept (the value of *Y* when all other parameters are set to 0), *B_1_,…,B_n_* are the regression coefficients of the independent variables *X_1_,…,X_n_* and *e* is the model error. In calculating *B_0_,…,B_n_,* the regression analysis ensures the maximal prediction of the dependent variable from the set of independent variables and the smallest overall model error *e*. This calculation is performed using the least squares method. 

The construction of a multiple regression model to assess the dependence of peak scores on the presence of significant IUPAC motifs in them for all ten ChIP-seq experiments was carried out using STATISTICA (StatSoftTM, Tulsa, OK, USA) with the “Multiple regression” option. 

In this case, the value of the natural logarithm of ChIP-seq peak significance (ln(PS)) was considered as the predicted dependent variable Y, and information about the presence or absence of the corresponding n IUPAC motifs in a particular sequence was considered as the predictors *X_1_,...,X_n_*. If the i-th motif was present in a particular peak sequence, then *X_i_* = 1, otherwise *X_i_* = 0. For example, for n = 3 motifs, of which only the first and third motifs are present in the j-th sequence of the set-in question, the predicted value is

*ln(PS)_j_ = y_j_= B*_0_ + *B*_1_ × 1 + *B*_2_ × 0 + *B*_3_ × 1 = *B*_0_ + *B*_1_ + *B*_3_.

For each of the training sets (Table 4) obtained in 10 ChIP-seq experiments, multiple regression models were built on the basis of information about the presence in the peak sequences of this set of n significant IUPAC motifs previously revealed in this set. For each of the 10 regression models obtained in this way, their predictive power was assessed both on the training sets of peak sequences and on the corresponding control sets (Table 4), by calculating the r values of the correlation between the predicted values of ln(PS) and those observed during the ChIP-seq experiment. 

It can be noted that this approach is quite universal, and not only data on the presence/absence of n motifs in sequences but also other information can be used as predictors. Using data from a number of ChIP-seq experiments, we, in particular, assessed the effectiveness of using information about the quantitative representation of each of the motifs, as well as the magnitude of their significance, as predictors. It turned out that these predictors were characterized by slightly worse prediction quality and lower correlation values r on the training and control sets. 

### 4.3. Construction of a Tree of Contextual Similarity of Motifs

A contextual similarity tree of the most significant IUPAC motifs detected in ChIP-seq peak sequences coming from the experiment with the transcription factor FOXA2 was constructed using the web system STAMP [122]. The Kullback–Leibler distance (the “KL” option) was used as a distance measure. The tree was visualized using the MEGA-X system [129]. 

### 4.4. Building a Heat Map for the Correlations of the Co-Occurrence of the Motifs

The heat map for the correlations of the co-occurrence of the most significant IUPAC motifs in the ChIP-seq peak sequences obtained from the experiment with the transcription factor FOXA2 was visualized using the web service Heatmapper [121]. Different shades of yellow correspond to different levels of positive correlation; blue, negative correlation; and white, non-significant correlation. 

### 4.5. Functional Annotation of the Motifs

Functional annotation of the most significant IUPAC motifs detected in the ChIP-seq peak sequences coming from the experiment with the target transcription factor FOXA2 was performed using the Tomtom system from the MEME Suite web package [38]. The motifs detected were compared with the PWMs contained in the JASPAR [34] and HOCOMOCO [33] databases with standard parameters. In the first stage of this procedure, the motifs that had significant similarity (*p* < 0.001) with the PWMs of the target transcription factor FOXA2 contained in either of the JASPAR [34] or HOCOMOCO [33] were identified. The IUPAC motifs thus obtained were considered target motifs. The remaining motifs did not have significant similarity with the PWMs of the target TF and were considered partner motifs. For all partner motifs, their similarity with the position–weight matrices of all known TFs contained in the JASPAR [34] and HOCOMOCO [33] databases was assessed. A partner motif was considered to be significantly similar to the transcription factor’s position–weight matrix if it had a significant (*p* < 0.001) similarity with the position–weight matrices of this TF in both databases, JASPAR [34] and HOCOMOCO [33]. 

### 4.6. Shuffling in Assessing the Significance of the Correlation between the Motifs Corresponding to the Binding Sites of the Target Motifs and the Motifs Corresponding to the Binding Sites of the Partner Transcription Factors

The assessment of the statistical significance of the correlations between the target motifs and partner motifs was carried out using the shuffling procedure. During this procedure, 10,000 random sets were generated by shuffling the nucleotides within each sequence of the given set of ChIP-seq peak sequences. For each generated set of sequences, the abundance, F, of each of the motifs considered was assessed. Next, the motifs, whose abundance F in the set of real ChIP-seq peak sequences exceeded their abundance in 95% of sets of randomly generated sequences, were selected. With the remaining significant IUPAC motifs for each generated random set, the correlation coefficient of the co-occurrence of the motifs corresponding to the binding sites of the target TF and the motifs corresponding to the binding sites of the partner TFs was assessed using the phi-coefficient [120]. Finally, the proportion of the correlation coefficients that were obtained from random sets and exceeded the absolute value of the correlation coefficient r calculated for the real set of ChIP-seq peak sequences was estimated and taken as the *p*-value. 

## 5. Conclusions

In the present work, we analyzed the contextual organization of ChIP-seq peak sequences in experiments with 10 TFs belonging to the six different superclasses categorized according to the types of their DNA-binding domains [99]. Using the original de novo motif discovery method Argo_CUDA [100], we identified both sets of significant IUPAC motifs corresponding to the target TF binding sites studied in each experiment and specific sets of motifs corresponding to the binding sites of the partner TFs in the peak DNA sequences revealed in each ChIP-seq experiment. Unlike heuristic methods, Argo_CUDA evaluates the significance of all possible IUPAC motifs of a given length, which guarantees finding a global optimum. Our analysis of the ChIP-seq data from the experiment with TF FOXA2 revealed a significant correlation between the presence of the target motifs corresponding to the binding sites for TF FOXA2 and the partner motifs corresponding to the binding sites for TF HNF4G. In the scientific literature, we found experimental evidence for a synergistic interaction between FOXA2 and transcription factors from the HNF4 family, which can explain this correlation. For all the ChIP-seq experiments considered, multiple regression models were constructed, demonstrating a significant dependence of the ChIP-seq peak sequence scores on the presence of sets of specific IUPAC motifs in these sequences. It has been shown that the most significant target motifs make a substantial contribution to the observed dependence. At the same time, the prediction quality can be improved through the use of less significant motifs as well as partner motifs. The contextual features of the ChIP-seq peaks that we have identified can be used to set up experiments aimed at testing potential partner interactions of TFs, the motifs of which are reliably co-represented in the sequences of ChIP-Seq peaks and also help in building potential regulatory gene networks involved in subtle developmental processes and tissue-specific gene expression. In addition, the significant IUPAC motifs we identified can be used to develop new methods for predicting the localization of potential TFBSs in genomic sequences. Unfortunately, despite the fact that we showed a highly reliable dependence of the peak score values on the presence of IUPAC motifs in ChIP-seq sequences, the correlation coefficient r we obtained did not exceed 0.57. This suggests that, to more effectively predict the peak score value, it will be necessary to use additional information, for example, about the relative position of motifs and their orientation in the sequences [130].

## Figures and Tables

**Table 1:** Brief description of ChIP-seq data analyzed in this work.

TF ^1^	ID ^2^	N ^3^	N_L_ ^4^	N_C_ ^5^	TF Superclass ^6^	TF Family ^7^	Cells Type ^8^	Ref. ^9^
CEBPA	39908	33,559	5000	28,559	1. Basic domains	1.1.8. CEBP-related	Mouse liver cells	[11]
CEBPB	72841	13,374	5000	8374	1. Basic domains	1.1.8. CEBP-related	Mouse liver cells	[102]
NFE2L2	70563	27,065	5000	22,065	1. Basic domains	1.1.1. Jun-related	Mouse bone marrow-derived macrophages	[12]
SP1	47755	24,404	5000	19,404	2. Zinc-coordinating DNA-binding domains	2.3.1. Three-zinc finger Krüppel-related	Mouse embryonic stem cells	[13]
GATA1	46419	7534	5000	2534	2. Zinc-coordinating DNA-binding domains	2.2.1. C4-GATA-related	Mouse erythroid cells of fetal liver	[103]
FOXA2	3266	25,191	5000	20,191	3. Helix-turn-helix domains	3.3.1. FOX	Mouse liver cells	[104]
FOXO1	92461	11,433	5000	6433	3. Helix-turn-helix domains	3.3.1. FOX	Mouse liver cells	[105]
NFYA	48618	5975	5000	975	4. Other all-alpha-helical DNA-binding domains	4.2.1. Heteromeric CCAAT-binding	Mouse embryonic stem cells	[14]
MEF2D	38097	34,789	5000	29,789	5. Alpha-Helices exposed by beta-structures	5.1.1. Regulators of differentiation	Mouse differentiating myoblasts C2C12	[15]
STAT5B	5839	18,510	5000	13,510	6. Immunoglobulin fold	6.2.1. STAT	Mouse liver cells	[106]

^1^ Name of the transcription factor; ^2^ ID of ChIP-seq experiment in the CistromeDB database [27]; ^3^ Total number of peaks identified in the ChIP-seq experiment and checked for the absence of non-canonical symbols; ^4^ Number of ChIP-seq peak DNA sequences included in the training set; ^5^ Number of ChIP-seq peak DNA sequences included in the control set; ^6^ Number and name of the superclass to which the TF belongs according to Wingender’s classification [99]; ^7^ Number and name of the family to which the TF belongs according to Wingender’s classification [99]; ^8^ Organism and cell type used in the ChIP-seq experiment; ^9^ Link to relevant publication.

**Table 2:** Characteristics of significant IUPAC motifs identified in the ChIP-seq peak DNA sequences for ten target transcription factors. Information is provided both on all motifs detected in each IUPAC motif set (*p*_0_ = 10^−2^) and on the most significant of them (*p*_0_ = 10^−30^).

Transcription Factor	Maximum Significance of the Motifs, −*log*_10_(*P_Bonf_* (*n*,*N*))	Number of Motifs Identified at *P_Bonf_* (*n*,*N*) < *p*_0_ = 10^−2^	Number of Motifs Identified at *P_Bonf_* (*n*,*N*) < *p*_0_ = 10^−30^
CEBPA	1585	151	36
CEBPB	1028	164	39
NFE2L2	880	239	71
SP1	520	243	80
GATA1	932	168	31
FOXA2	1462	202	50
FOXO1	447	220	51
NFYA	1134	270	81
MEF2D	749	219	57
STAT5B	744	181	44

**Table 3:** Examples and characteristics of the most significant (*P_Bonf_* (*n*,*N*) < 10^−30^) IUPAC motifs detected with Argo_CUDA in the training set of ChIP-seq peak sequences in the experiment involving the transcription factor FOXA2.

Motif	Actual Abundance ^1^, F	Expected Abundance ^2^, Q	Significance ^3^, −*log*_10_(*P_Bonf_* (*n*,*N*))
TRTWKACH	0.67	0.15	1462
RTTKACHY	0.54	0.20	620
TMAAYANS	0.54	0.26	395
TWKACHYW	0.55	0.27	367
TTKRTYTW	0.30	0.14	172
TKAHYTWK	0.46	0.27	157
TRTTKRTY	0.30	0.15	139
RAAYHAAY	0.31	0.17	128
TTRNGHAA	0.29	0.16	116
KACDTWGN	0.30	0.17	103
TAAHYABW	0.32	0.19	91
ARMYAAGV	0.30	0.18	81
TGYGTACH	0.09	0.03	77
TRTWTGCW	0.15	0.07	69
AAAMAAAR	0.13	0.06	60
ATMMAYAN	0.28	0.18	55
WTRTTTGY	0.19	0.11	54
TCRAYADW	0.19	0.11	52
GTACRCAH	0.06	0.02	46
TTYGCTYW	0.12	0.06	45
TNGCTHWG	0.24	0.16	40
TNACYMWG	0.30	0.22	31

^1^ F is the proportion of sequences observed to contain at least one IUPAC motif; ^2^ Q is the proportion of sequences expected to contain the IUPAC motif for random reasons; ^3^ −*log*_10_(*P_Bonf_* (*n*,*N*)) is the Bonferroni-corrected binomial probability of observing motifs for random reasons (see Section 4).

**Table 4:** The results of assessing the dependence of the natural logarithm of peak scores (*ln(PS)*) on the presence of significant (*p* < 10^−2^) IUPAC motifs in the DNA sequences of the training and control sets for ChIP-seq experiments with 10 transcription factors. The multiple regression coefficients for the training sets were calculated using STATISTICA (StatSoft^TM^, Tulsa, OK, USA).

		Training Set			Control Set		
Transcription Factor	Number of Significant Motifs	Number of Sequences in the Set, N	Correlation Coefficient, r	Max *ln(PS)*	Number of Sequences in the Set, N	Correlation Coefficient, r	Max *ln(PS)*
CEBPA	151	5000	0.38	5.29	28,559	0.11	3.6
CEBPB	164	5000	0.37	5.53	8374	0.13	2.74
NFE2L2	239	5000	0.33	5.54	22,065	0.01 *	3.17
SP1	243	5000	0.57	5.69	19,404	0.06	3.5
GATA1	168	5000	0.3	5.45	2534	−0.03 *	2.42
FOXA2	202	5000	0.33	5.4	20,191	0.09	3.49
FOXO1	220	5000	0.32	5.68	6433	0.08	2.45
NFYA	270	5000	0.53	5.46	975	0.07	1.88
MEF2D	219	5000	0.36	5.41	29,789	0.07	2.93
STAT5B	181	5000	0.32	5.2	13,510	0.09	3.1

* Non-significant (*p* > 0.05) correlation coefficients r in assessing the relationship between the predicted and observed values of peak scores, with the Bonferroni correction [119] taken into account.

**Table 5:** Examples of independent variables with significant (*p* < 0.05) regression coefficients obtained when constructing a multiple regression model to assess the dependence of the *ln*(*PS*) value in the ChIP-seq experiment with the transcription factor FOXA2 on the presence of IUPAC motifs in these peaks. Multiple regression coefficients were calculated using STATISTICA (StatSoft^TM^, Tulsa, OK, USA).

Independent Variable	Regression Coefficient	*p*-Value
TRTWKACH	0.064945	0.000003
RTTKACHY	0.027893	0.039629
TMAAYANS	0.057817	0.0000001
TWKACHYW	0.027454	0.006242
TTRNGHAA	0.024021	0.031108
KACDTWGN	0.028459	0.011256
TAAHYABW	−0.027147	0.006997
CCRCCCCB	−0.081117	0.000936
CGBTCGVN	0.118823	0.006146
TTSGYWRN	0.018182	0.040455
AACAWGVV	0.032637	0.004226
TRATTRRY	0.037430	0.032885
TTNRTTCW	−0.035167	0.016293
GGWGRVHG	0.024464	0.008203
WSCSTRKS	0.018255	0.033926
HCGBTCGV	−0.113689	0.043413
GGCRGGAV	0.046776	0.009803
TTKACWRA	0.033587	0.035103
GGHNGAGH	0.021662	0.040533
KRAGCBAN	0.026475	0.009812
ACVCWRMS	0.023771	0.010771
WCCCCVVC	0.04015	0.01686
AMVCAYAG	0.03683	0.00944
CGNMYCGG	0.08602	0.008174
CKTCCGKN	0.0739	0.044389
CCTSGRMK	0.036704	0.015545
TGTGGACW	0.103851	0.000909
GSARHGGR	−0.023597	0.040487
WGCGGYSG	0.084659	0.021616
TWWKTAAY	−0.053886	0.001453

**Table 6:** Examples of independent variables with significant (*p* < 0.05) regression coefficients obtained when constructing a multiple regression model to assess the dependence of the *ln(PS)* value in the ChIP-seq experiment with the transcription factor FOXA2 on the abundance of the most significant (*P_Bonf_* (*n*,*N*) < 10^−30^) IUPAC motifs. Multiple regression coefficients were calculated using STATISTICA (StatSoft^TM^, Tulsa, OK, USA).

Independent Variable	Regression Coefficient	*p*-Value
TRTWKACH	0.066537	0.000001
TMAAYANS	0.051297	0.0000001
TWKACHYW	0.021614	0.028887
KACDTWGN	0.025535	0.015482
TAAHYABW	−0.033779	0.000422
ARMYAAGV	0.02232	0.020651
CCRCCCCB	−0.056879	0.017154
CGSCBBCG	0.040932	0.014188
GCGYKCGN	0.115472	0.009763
CGMRCGCV	−0.131678	0.009819
ANCRAHGV	0.018453	0.040805

**Table 7:** Examples of independent variables with significant (*p* < 0.05) regression coefficients obtained when constructing a multiple regression model to assess the dependence of the ln(PS) value in the ChIP-seq experiment with the transcription factor SP1 on the abundance of the most significant (*P_Bonf_* (*n*,*N*) < 10^−30^) IUPAC motifs. Multiple regression coefficients were calculated using STATISTICA (StatSoft^TM^, Tulsa, OK, USA).

Independent Variable	Regression Coefficient	*p*-Value
ATTSGHYR	0.06863	0.009941
RRCSAATS	−0.074581	0.021391
TGTAGTYY	0.25583	0.0000001
CSAATSRV	0.141722	0.0000001
TACAWNTC	−0.103949	0.031145
ANWTGTAG	0.155459	0.000388
TBYBATTG	0.05046	0.00207
GGGANWTG	0.147136	0.0000001
WGGGYGGG	0.042234	0.038834
TKCYGGGW	0.051412	0.00338
CTTCCKGB	−0.046938	0.011891
RGGCGGGH	0.050394	0.001738
ATTSGYYY	0.076936	0.00512
TATTGGHY	0.172836	0.000022
GGGHSGWG	−0.025723	0.047439
CGGKRCBD	0.029378	0.019498
RABBGACR	0.075836	0.0000001
TTGGTCNR	0.079731	0.000773
TAGTYYWH	0.050178	0.015284
TTTRHWTW	−0.037338	0.037889
WKCAAAKN	−0.043257	0.004714
GTCAYGTG	−0.098339	0.001426
TGANTGAC	0.134395	0.000061
AVHGAYAR	−0.043841	0.001981
AYGATTSG	0.242444	0.0000001
GGATTSGH	0.121602	0.000005
ACGSAHGY	0.053019	0.026689
GVATKCTG	0.058493	0.038876
AGATAAGV	−0.129716	0.000021

**Table 8:** Examples of independent variables with significant (*p* < 0.05) regression coefficients obtained when constructing a multiple regression model to assess the dependence of the *ln(PS)* value in the ChIP-seq experiment with the transcription factor FOXA2 on the presence of the most significant (*P_Bonf_* (*n*,*N*) < 10^−30^) target IUPAC motifs. Multiple regression coefficients were calculated using STATISTICA (StatSoft^TM^, Tulsa, OK, USA).

Independent Variable	Regression Coefficient	*p*-Value
TRTWKACH	0.055717	0.000001
RTTKACHY	0.027913	0.02056
TMAAYANS	0.047318	0.000001
TWKACHYW	0.019684	0.04549
TTKRTYTW	−0.02034	0.046044
KACDTWGN	0.020031	0.035274
TAAHYABW	−0.036249	0.000121
ARMYAAGV	0.019283	0.037902
AAAMAAAR	−0.009348	0.024866

**Table 9:** Results of the annotation of partner motifs detected in the ChIP-seq peak sequences from the **FOXA2_L_** set.

Partner TF ^1^	Number of Motifs ^2^	TF Family ^3^	TF Subfamily ^4^
HNF4G	6	2.1.3. RXR-related receptors (NR2)	2.1.3.2. HNF-4 (NR2A)
SP1	6	2.3.1. Three-zinc finger Krüppel-related factors	2.3.1.1. SP1-like factors
FOXO3	2	3.3.1. Forkhead box (FOX) factors	3.3.1.15. FOXO
POU5F1	2	3.1.10. POU domain factors	3.1.10.5. POU5 (OCT-3/4-like factors)
NR2F1	1	2.1.3. RXR-related receptors (NR2)	2.1.3.5. COUP-like receptors (NR2F)
ZNF148	1	2.3.3. More than 3 adjacent zinc finger factors	2.3.3.13. ZNF148-like factors
EGR1	1	2.3.1. Three-zinc finger Krüppel-related factors	2.3.1.3. EGR factors
NFYC	1	4.2.1. Heteromeric CCAAT-binding factors	4.2.1.0.3. NF-YC
SOX2	1	4.1.1. SOX-related factors	4.1.1.2. Group B
ZBTB14	1	2.3.3. More than 3 adjacent zinc finger factors	2.3.3.0. unclassified
MAFG	1	1.1.3. MAF-related factors	1.1.3.2. Small Maf factors

^1^ Name of the partner transcription factor; ^2^ Number of motifs significantly (*p* < 0.001) similar to the position–weight matrix of the transcription factor in the JASPAR and HOCOMOCO databases, according to the Tomtom [123] annotation; ^3^ Number and name of the family to which the TF belongs, according to Wingender’s classification [99]; ^4^ Number and name of the subfamily to which the TF belongs, according to Wingender’s classification [99].

**Table 10:** Correlation coefficients obtained using the phi-coefficient to assess the interdependencies of the localization of the motifs corresponding to the binding sites of the partner transcription factors and the motifs corresponding to the binding sites of the target TF FOXA2 on the training set **FOXA2_L_** of ChIP-seq peak sequences. Significant (*p* < 0.05) correlation coefficients are asterisked.

Partner TF	Correlation Coefficient, r
HNF4G	0.116739 *
SP1	−0.12075
FOXO3	0.106967
POU5F1	0.076699
NR2F1	−0.02758
ZNF148	−0.06076
MAFG	0.106967 *

* Significant (*p* < 0.05) correlation coefficients.

**Table 11:** Experimental evidence for functional interactions between FOXA2 and other partner transcription factors.

Partner TF	Gene or Genomic Regions	Summary	Reference
HNF4 subfamily	Human APOB enhancer	Cooperative interaction between FOXA2 and HNF4 in mediating enhancer function	[124]
HNF4 subfamily (HNF4A)	DNA regions from HepG2 ChIP-seq for FOXA2 and HNF4A	ChIP-sequencing revealed that FOXA2 peaks were co-localizing with HNF4A peaks	[125]
HNF4 subfamily (HNF4A)	DNA regions from adult mouse liver or embryonic hepatoblasts ChIP-seq for FOXA2 and HNF4A	ChIP-sequencing revealed that FOXA2 peaks were co-localizing with HNF4A peaks	[126]
HNF4 subfamily (HNF4A)	Human F2 enhancer	FOXA2 and HNF4A were found to be bound to the enhancer of this gene	[127]
HNF4 subfamily (HNF4A)	DNA regions from adult mouse liver ChIP-seq for FOXA2 and HNF4A	ChIP-sequencing revealed that FOXA2 peaks were co-localizing with HNF4A peaks	[128]

**Table 12:** Binary representation and hash-coding (hashing) of the 15-letter IUPAC code.

IUPAC Letter	A	T	G	C	R	Y	M	K	W	S	B	H	V	D	N
Nucl. Variants	A	T	G	C	G/A	T/C	A/C	G/T	A/T	C/G	Not A	Not G	Not T	Not C	N
**A**	1	0	0	0	1	0	1	0	1	0	0	1	1	1	1
**T**	0	1	0	0	0	1	0	1	1	0	1	1	0	1	1
**G**	0	0	1	0	1	0	0	1	0	1	1	0	1	1	1
**C**	0	0	0	1	0	1	1	0	0	1	1	1	1	0	1
**Hash**	0001	0010	0100	1000	0101	1010	1001	0110	0011	1100	1110	1011	1101	0111	1111

## Data Availability

Links to the analyzed data obtained from the CistromeDB database are given in Table 1. Section 2.1 is devoted to a description of this data.

## Supplementary Materials

The supporting information can be downloaded at: https://www.mdpi.com/article/10.3390/ijms25021011/s1.

## Abbreviations

CE	Composite element
ChIP-seq	Chromatin immunoprecipitation followed by sequencing
CRM	Cis-regulatory module
DNA	Deoxyribonucleic acid
GPU	Graphics processing unit
HMM	Hidden Markov model
PS	Peak score
PWM	Position–weight matrix
TF	Transcription factor
TFBS	Transcription factor binding site
TFFM	Transcription factor flexible model
