# Supplementary material for: Peak Scores Significantly Depend on the Relationships between Contextual Signals in ChIP-Seq Peaks
Source: Int J Mol Sci. 2024 Jan 13;25(2):1011. doi: 10.3390/ijms25021011 (PMC10816497; doi:10.3390/ijms25021011)
Supplement: Supplementary file 1 [file ijms-25-01011-s001.zip › Supplementary Matherials.pdf]

International Journal of *Molecular Sciences*

MDPI

*Article*

# Peak Scores Significantly Depend on the Relationships between Contextual Signals in ChIP-Seq Peaks

**Oleg Vishnevsky [1,2,*], Andrey Bocharnikov [2] and Elena Ignatieva [1,2]**

[1] Institute of Cytology and Genetics, 630090 Novosibirsk, Russia; eignat@bionet.nsc.ru
[2] Department of Natural Science, Novosibirsk State University, 630090 Novosibirsk, Russia; andrey.bocharnikov@gmail.com
* Correspondence: oleg@bionet.nsc.ru

**Table S1.** Independent variables obtained when constructing a multiple regression model to assess the dependence of the *ln(PS)* value in the ChIP-Seq experiment with the FoxA2 transcription factor on the presence of IUPAC motifs in these peaks. Multiple regression coefficients were calculated using the Statistica system (Statsoft™, Tulsa, USA).

| Independent variable | The value of the regression coefficient | p - value |
|---|---|---|
| TRTWKACH | 0.064945 | 0.000003 |
| RTTKACHY | 0.027893 | 0.039629 |
| TMAAYANS | 0.057817 | 0 |
| TWKACHYW | 0.027454 | 0.006242 |
| TTKRTYTW | -0.0183 | 0.106331 |
| TKAHYTWK | 0.004714 | 0.556024 |
| TRTTKRTY | 0.022418 | 0.110034 |
| GRMCTHNG | 0.003873 | 0.669797 |
| RAAYHAAY | -0.00456 | 0.71945 |
| TTRNGHAA | 0.024021 | 0.031108 |
| KACDTWGN | 0.028459 | 0.011256 |
| GGGGHGGG | 0.020249 | 0.464922 |
| GKDCRNAG | -0.00612 | 0.507082 |
| RAGKYCAN | 0.013327 | 0.267003 |
| GGGHGGGG | -0.00193 | 0.941753 |
| TAAHYABW | -0.02715 | 0.006997 |
| ARMYAAGV | 0.014113 | 0.179725 |
| TGYGTACH | -0.00186 | 0.951609 |
| RTWKACHR | -0.0171 | 0.166124 |
| AGKTCRND | -0.01803 | 0.149243 |
| TRTWTGCW | 0.033065 | 0.070134 |
| CCRCCCCB | -0.08112 | 0.000936 |
| TGNMCTYW | -0.00413 | 0.67892 |
| ACBTWGNH | -0.00567 | 0.522861 |
| TAYRCARN | 0.004306 | 0.741284 |
| AAAMAAAR | -0.004 | 0.379818 |
| CGSCBBCG | 0.003226 | 0.874502 |

| | | |
|---|---|---|
| VTTNGCTN | -0.00846 | 0.398943 |
| ATMMAYAN | -0.01157 | 0.34691 |
| WTRTTTGY | -0.02029 | 0.214387 |
| TCRAYADW | 0.021752 | 0.090305 |
| GGYGGSGS | -0.02072 | 0.314472 |
| GTACRCAH | 0.004619 | 0.897252 |
| TTYGCTYW | 0.025579 | 0.163475 |
| GCGGNGCG | 0.04153 | 0.288321 |
| TDCHTAAB | -0.01677 | 0.238398 |
| TNGCTHWG | -0.00426 | 0.709402 |
| RRGRCRGR | -0.0052 | 0.587477 |
| GCGYKCGN | 0.010204 | 0.901432 |
| CGMRCGCV | -0.07895 | 0.325373 |
| RSDYRGTG | 0.013789 | 0.15695 |
| GYMCTNBG | 0.013578 | 0.137178 |
| AAMYAYBR | -0.00129 | 0.885633 |
| GGVVRWGG | 0.001835 | 0.831654 |
| ARYBAATB | 0.002026 | 0.871641 |
| ACWVAGMW | 0.006341 | 0.498484 |
| TTKTTTTS | -0.01969 | 0.297221 |
| TNACYMWG | -0.01787 | 0.05649 |
| AMCTYWGN | 0.005755 | 0.537538 |
| ANCRAHGV | 0.018936 | 0.058036 |
| CGBTCGVN | 0.118823 | 0.006146 |
| CGGHGCGB | -0.03953 | 0.265681 |
| GTGTACWY | 0.054044 | 0.060788 |
| YTGYCYTN | 0.014752 | 0.116004 |
| TTSGYWRN | 0.018182 | 0.040455 |
| GGGCNGGG | -0.00008 | 0.997041 |
| MYTMACYN | -0.00111 | 0.915654 |
| CKTYGGHN | 0.004876 | 0.729611 |
| TCSCKHCS | -0.01649 | 0.333777 |
| AKAYVAAY | -0.00738 | 0.617441 |
| AACAWGVV | 0.032637 | 0.004226 |
| WRCCBTDG | -0.00361 | 0.733808 |
| GCGDCSGS | 0.001905 | 0.952881 |
| CTYRGMMB | 0.007255 | 0.436816 |
| AAAAAAAT | -0.04381 | 0.229676 |
| ATTKCDHA | 0.020283 | 0.151477 |
| TRATTRRY | 0.03743 | 0.032885 |
| DGGRKGMG | -0.01986 | 0.105866 |
| TTNRTTCW | -0.03517 | 0.016293 |

| | | |
|---|---|---|
| CCGNMKCS | 0.034576 | 0.050553 |
| GCAHRCRS | -0.01072 | 0.389983 |
| TNCKWATB | 0.020401 | 0.142042 |
| GGWGRVHG | 0.024464 | 0.008203 |
| ATYGATYR | -0.01486 | 0.552127 |
| AWGCVABS | -0.00066 | 0.952915 |
| WSCSTRKS | 0.018255 | 0.033926 |
| YCWCYGWV | 0.004817 | 0.601776 |
| CWMYGTNR | 0.001395 | 0.880217 |
| ASSSAGGV | 0.001651 | 0.881211 |
| CGYCGCSB | -0.00872 | 0.756327 |
| CAWACAAR | 0.009464 | 0.60626 |
| GGHYRGGG | -0.00534 | 0.729401 |
| AARWRCAA | -0.00721 | 0.635604 |
| ATBATTAA | -0.0153 | 0.680502 |
| GHGGKMGG | 0.011976 | 0.514792 |
| AGWCBWYG | 0.012495 | 0.440684 |
| AATYATTA | -0.00379 | 0.931667 |
| WMHTAACY | -0.01141 | 0.500393 |
| GNCWCYGH | 0.010461 | 0.243576 |
| HTAACCHB | 0.008789 | 0.565996 |
| ARKSSAAA | 0.021976 | 0.060556 |
| CBWATCDV | 0.014192 | 0.266432 |
| CTRSGHWB | 0.000268 | 0.975205 |
| GBCKTDGB | 0.015655 | 0.131419 |
| CGVRCGGS | 0.006716 | 0.808555 |
| KGKCMYYG | 0.003218 | 0.760706 |
| TGSMTDCB | -0.00391 | 0.701575 |
| TTGCDWCA | 0.019258 | 0.355813 |
| HCGBTCGV | -0.11369 | 0.043413 |
| CSWVGCTV | -0.01206 | 0.289738 |
| GGCRGGAV | 0.046776 | 0.009803 |
| TGWMCCYW | -0.00206 | 0.885991 |
| ATTKCRWC | -0.01219 | 0.646997 |
| TNSVDTTC | -0.00034 | 0.968723 |
| ACDWGGHH | 0.001697 | 0.856515 |
| GCGMCGMG | 0.007786 | 0.864423 |
| TTKACWRA | 0.033587 | 0.035103 |
| DAHGAAAR | -0.00644 | 0.657093 |
| GCBWAKGR | -0.00908 | 0.560534 |
| GGHNGAGH | 0.021662 | 0.040533 |
| KRAGCBAN | 0.026475 | 0.009812 |

| | | |
|---|---|---|
| DRGRMAAG | -0.00716 | 0.502581 |
| YRTCKHYS | -0.00863 | 0.391891 |
| TTTGWATY | 0.011182 | 0.676263 |
| HCCWWCCS | 0.007283 | 0.643497 |
| ACVCWRMS | 0.023771 | 0.010771 |
| TGGGGNKG | -0.0204 | 0.244909 |
| DRATKTCR | -0.00083 | 0.95523 |
| GGCSMCGC | 0.00678 | 0.837769 |
| CRTGCGCR | 0.047644 | 0.191893 |
| WCCCCVVC | 0.04015 | 0.01686 |
| TCKWYCYN | -0.00397 | 0.711223 |
| YRGKTCRB | 0.017374 | 0.224318 |
| SRCWCAGN | -0.01564 | 0.099753 |
| AMVCAYAG | 0.03683 | 0.00944 |
| CTMACTVN | -0.01458 | 0.330524 |
| CGNMYCGG | 0.08602 | 0.008174 |
| TACRCAYD | -0.00512 | 0.811333 |
| RTTYGMAY | 0.029037 | 0.123496 |
| CKTCCGKN | 0.0739 | 0.044389 |
| YKCKTBCB | 0.010328 | 0.27771 |
| TMAYYTYB | -0.00848 | 0.370015 |
| AGCGYKCG | 0.039415 | 0.620771 |
| RCCHMGTG | 0.021349 | 0.190224 |
| WTWCAAAR | 0.005842 | 0.734245 |
| AWCVSWGM | 0.008588 | 0.361091 |
| KNGMAAYG | -0.00087 | 0.947455 |
| CTTBGWTS | 0.00698 | 0.701813 |
| CTBCSTSK | -0.01883 | 0.096703 |
| KGKGCGTG | -0.00189 | 0.95178 |
| GTBCMHGG | 0.020722 | 0.098363 |
| MCGGAMGK | -0.04252 | 0.372464 |
| WNGRATKR | -0.01159 | 0.250609 |
| RWCGBTCG | -0.07535 | 0.206749 |
| TCCWSSYC | 0.02072 | 0.092817 |
| TDCKGWTK | -0.00501 | 0.682396 |
| RCTCYGDV | 0.013253 | 0.171227 |
| GGKTCRKD | -0.00751 | 0.660859 |
| ACRMRCAC | -0.00527 | 0.19023 |
| DNCKCACB | 0.01557 | 0.191313 |
| RATMTTTG | 0.020472 | 0.335884 |
| KTTCWBTK | -0.00235 | 0.802398 |
| ACATAGHH | -0.00382 | 0.871105 |

| | | |
|---|---|---|
| MGGCKKGS | 0.020086 | 0.171724 |
| CCTSGRMK | 0.036704 | 0.015545 |
| TATCTWRK | 0.028604 | 0.304342 |
| MKCYACCV | -0.00645 | 0.688424 |
| TRCYMAGV | -0.00137 | 0.908218 |
| TTAWYTDA | 0.022748 | 0.241918 |
| GGCSGSCG | 0.026619 | 0.428897 |
| SRTGGTGS | -0.02642 | 0.24453 |
| AWGRAAAA | 0.004712 | 0.831298 |
| GMGBWGGG | -0.01411 | 0.397874 |
| RCSRATCG | -0.032 | 0.481291 |
| WCSGBBCG | 0.033847 | 0.149445 |
| GHRGNTGG | -0.00703 | 0.554474 |
| RCWCAGKM | 0.019598 | 0.114461 |
| TWTRTASW | -0.00697 | 0.69251 |
| TDCTYTTY | 0.006017 | 0.669828 |
| GAHBTCAY | 0.006527 | 0.660263 |
| TGTGGACW | 0.103851 | 0.000909 |
| YWRGAAYB | -0.00396 | 0.702522 |
| TGCAATSB | 0.026351 | 0.265777 |
| RGAAYTSR | -0.02954 | 0.069598 |
| GCTCRCYS | 0.032263 | 0.172095 |
| TBRCTMAY | -0.00666 | 0.63315 |
| KMCRAMGV | 0.004017 | 0.790012 |
| TGMMCYGW | -0.01006 | 0.522609 |
| TRGTTHTB | 0.000909 | 0.951084 |
| AMVYATVC | -0.00731 | 0.583863 |
| AAWTAAMR | 0.007168 | 0.68754 |
| TKMGTYAY | 0.023544 | 0.236221 |
| TTAAAAAW | -0.05005 | 0.119039 |
| AWTCGWTV | 0.033755 | 0.422023 |
| AGKYCABC | 0.010805 | 0.50076 |
| GTCCMCAD | -0.00069 | 0.97725 |
| TRGRAAAW | -0.00471 | 0.78652 |
| GSARHGGR | -0.0236 | 0.040487 |
| TKASACAK | 0.007172 | 0.670823 |
| KRATKAAR | -0.02418 | 0.163617 |
| WGCGGYSG | 0.084659 | 0.021616 |
| ATCGATHY | 0.026319 | 0.530421 |
| CKTGCKTV | -0.00029 | 0.989536 |
| TWWKTAAY | -0.05389 | 0.001453 |
| CGDSASCG | 0.04324 | 0.203914 |

| | | |
|---|---|---|
| RGAAAARH | -0.01602 | 0.309993 |
| GGWAGSNG | 0.023198 | 0.144094 |
| GCRGGMMG | -0.01102 | 0.575352 |
| GCTMAGCD | -0.00019 | 0.993453 |
| GCCCHSGM | -0.00688 | 0.685831 |
| AYAAGCDA | -0.00893 | 0.732848 |
| CGKKACGB | 0.028595 | 0.484864 |

**Table S2.** Independent variables obtained when constructing a multiple regression model to assess the dependence of the ln(PS) value in the ChIP-Seq experiment with the FoxA2 transcription factor on the presence of the most significant ($P_{Bonf}(n,N) < 10^{-30}$) IUPAC motifs. Multiple regression coefficients were calculated using Statistica (Statsoft™, Tulsa, USA).

| Independent variable | The value of the regression coefficient | p - value |
|---|---|---|
| TRTWKACH | 0.066537 | 0.000001 |
| RTTKACHY | 0.020576 | 0.127869 |
| TMAAYANS | 0.051297 | 0 |
| TWKACHYW | 0.021614 | 0.028887 |
| TTKRTYTW | -0.01798 | 0.087215 |
| TKAHYTWK | -0.00452 | 0.558784 |
| TRTTKRTY | 0.025957 | 0.056908 |
| GRMCTHNG | 0.007598 | 0.389525 |
| RAAYHAAY | -0.02022 | 0.079725 |
| TTRNGHAA | 0.014906 | 0.161514 |
| KACDTWGN | 0.025535 | 0.015482 |
| GGGGHGGG | 0.008683 | 0.743245 |
| GKDCRNAG | -0.00599 | 0.494705 |
| RAGKYCAN | 0.019199 | 0.075093 |
| GGGHGGGG | 0.00606 | 0.803012 |
| TAAHYABW | -0.03378 | 0.000422 |
| ARMYAAGV | 0.02232 | 0.020651 |
| TGYGTACH | 0.01832 | 0.50862 |
| RTWKACHR | -0.01418 | 0.219779 |
| AGKTCRND | -0.01949 | 0.05581 |
| TRTWTGCW | 0.021962 | 0.185892 |
| CCRCCCCB | -0.05688 | 0.017154 |
| TGNMCTYW | -0.00593 | 0.535821 |
| ACBTWGNH | -0.00828 | 0.3323 |
| TAYRCARN | 0.00643 | 0.603919 |
| AAAMAAAR | -0.00702 | 0.110747 |
| CGSCBBCG | 0.040932 | 0.014188 |
| VTTNGCTN | -0.00617 | 0.51208 |
| ATMMAYAN | -0.0153 | 0.197303 |

| | | |
|---|---|---|
| WTRTTTGY | -0.01027 | 0.452303 |
| TCRAYADW | 0.006622 | 0.598369 |
| GGYGGSGS | -0.01007 | 0.572261 |
| GTACRCAH | -0.00787 | 0.816082 |
| TTYGCTYW | 0.016563 | 0.35154 |
| GCGGNGCG | 0.032148 | 0.281616 |
| TDCHTAAB | -0.021 | 0.132216 |
| TNGCTHWG | 0.005806 | 0.58342 |
| RRGRCRGR | 0.009095 | 0.265196 |
| GCGYKCGN | 0.115472 | 0.009763 |
| CGMRCGCV | -0.13168 | 0.009819 |
| RSDYRGTG | 0.013772 | 0.127386 |
| GYMCTNBG | 0.01683 | 0.055736 |
| AAMYAYBR | -0.00362 | 0.679905 |
| GGVVRWGG | 0.000316 | 0.967446 |
| ARYBAATB | 0.007057 | 0.523718 |
| ACWVAGMW | 0.003306 | 0.705764 |
| TTKTTTTS | -0.03302 | 0.062233 |
| TNACYMWG | -0.01257 | 0.158219 |
| AMCTYWGN | -0.00054 | 0.952723 |
| ANCRAHGV | 0.018453 | 0.040805 |

**Table S3.** Independent variables obtained when constructing a multiple regression model to assess the dependence of the *ln(PS)* value in the ChIP-Seq experiment with the Sp1 transcription factor on the presence of IUPAC motifs in these peaks. Multiple regession coefficients were calculated using the Statistica system (Statsoft™, Tulsa, USA).

| Independent variable | The value of the regression coefficient | p - value |
|---|---|---|
| RCSAATSR | -0.03968 | 0.149248 |
| ATTSGHYR | 0.06863 | 0.009941 |
| RRCSAATS | -0.07458 | 0.021391 |
| GRGGYGGG | -0.00775 | 0.635139 |
| GGGYGGGG | -0.03714 | 0.052744 |
| WTGTAGTY | 0.0723 | 0.072885 |
| TGTAGTYY | 0.25583 | 0 |
| CSAATSRV | 0.141722 | 0 |
| GGCGGGRC | -0.02481 | 0.298582 |
| TACAWNTC | -0.10395 | 0.031145 |
| RKGGGYGG | 0.004386 | 0.762046 |
| ANWTGTAG | 0.155459 | 0.000388 |
| ACAWNTCC | -0.00684 | 0.845511 |
| TBYBATTG | 0.05046 | 0.00207 |
| GGGANWTG | 0.147136 | 0 |

| | | |
|---|---|---|
| TTGGYTRN | 0.025576 | 0.139164 |
| RCWTMCKG | 0.018955 | 0.303513 |
| GCGGGRCB | -0.02321 | 0.306679 |
| CGSAHGYG | -0.01685 | 0.31289 |
| AWYTCCCA | 0.007676 | 0.782633 |
| GCGCABGC | 0.029531 | 0.178375 |
| AATBARMD | -0.01655 | 0.225488 |
| WGGGYGGG | 0.042234 | 0.038834 |
| WRGGGGYG | 0.002944 | 0.852301 |
| GGARGBGG | 0.014922 | 0.23332 |
| TKCYGGGW | 0.051412 | 0.00338 |
| CTTCCKGB | -0.04694 | 0.011891 |
| RGGCGGGH | 0.050394 | 0.001738 |
| ATTSGYYY | 0.076936 | 0.00512 |
| AABATGGC | 0.043041 | 0.320513 |
| TATTGGHY | 0.172836 | 0.000022 |
| GGGHSGWG | -0.02572 | 0.047439 |
| TRCGCAYG | -0.05127 | 0.104776 |
| RTGACGTM | -0.02342 | 0.553568 |
| TTCCKRTY | 0.008231 | 0.68229 |
| GGGAGGGG | -0.04261 | 0.127127 |
| TGWCGTMA | -0.00789 | 0.86545 |
| CGGKRCBD | 0.029378 | 0.019498 |
| RCSAATAG | -0.02511 | 0.61291 |
| ACGWCAYB | -0.00523 | 0.871806 |
| GGCSGWRC | -0.01065 | 0.5993 |
| GGGGAGGG | -0.02574 | 0.33396 |
| GKAAGTRH | -0.00409 | 0.833201 |
| WHTCCCAG | 0.008707 | 0.67101 |
| ATKCTGGG | -0.01669 | 0.657205 |
| CSAATAGV | 0.020195 | 0.642444 |
| RGTGGGBG | -0.02822 | 0.097165 |
| GCAVGCGC | 0.025662 | 0.302115 |
| GADSGMGG | 0.025278 | 0.051817 |
| TCSRCCCC | -0.01803 | 0.424184 |
| KGGGCGTG | 0.001341 | 0.964425 |
| RABBGACR | 0.075836 | 0 |
| TCYRGGWH | 0.005942 | 0.660425 |
| CAMTCAVS | -0.03009 | 0.102829 |
| GGGCGTGG | -0.05943 | 0.169547 |
| TRATTSGA | 0.01216 | 0.785801 |
| GATTSGAY | -0.03686 | 0.437022 |

| | | |
|---|---|---|
| AGGNNGGG | -0.01015 | 0.365549 |
| AHDCRMAB | 0.004712 | 0.53238 |
| DACTTCCG | 0.003082 | 0.903334 |
| TTGGTCNR | 0.079731 | 0.000773 |
| TAGTYYWH | 0.050178 | 0.015284 |
| TSGMTRHB | -0.0117 | 0.299792 |
| TTTRHWTW | -0.03734 | 0.037889 |
| WKCAAAKN | -0.04326 | 0.004714 |
| GGWGGGGH | -0.02704 | 0.124095 |
| ATGGCRRC | -0.01606 | 0.556384 |
| CCATSTTK | -0.02915 | 0.444397 |
| GTCAYGTG | -0.09834 | 0.001426 |
| TGANTGAC | 0.134395 | 0.000061 |
| AVHGAYAR | -0.04384 | 0.001981 |
| GGCGTGGY | -0.00237 | 0.94732 |
| ABATGGCG | -0.0142 | 0.753224 |
| GCSGWGCB | 0.029308 | 0.078454 |
| AYGATTSG | 0.242444 | 0 |
| GGATTSGH | 0.121602 | 0.000005 |
| ACGSAHGY | 0.053019 | 0.026689 |
| GVATKCTG | 0.058493 | 0.038876 |
| AGATAAGV | -0.12972 | 0.000021 |
| CGCAGGCG | -0.0377 | 0.310418 |

**Table S4.** Independent variables obtained when constructing a multiple regression model to assess the dependence of the ln(PS) value in the ChIP-Seq experiment with the FoxA2 transcription factor on the presence of the most significant ($P_{Bonf}(n,N) < 10^{-30}$) target IUPAC motifs. Multiple regression coefficients were calculated using Statistica (Statsoft™, Tulsa, USA).

| Independent variable | The value of the regression coefficient | p - value |
|---|---|---|
| TRTWKACH | 0.055717 | 0.000001 |
| RTTKACHY | 0.027913 | 0.02056 |
| TMAAYANS | 0.047318 | 0.000001 |
| TWKACHYW | 0.019684 | 0.04549 |
| TTKRTYTW | -0.02034 | 0.046044 |
| TKAHYTWK | -0.00736 | 0.313546 |
| TRTTKRTY | 0.025064 | 0.063592 |
| RAAYHAAY | -0.02213 | 0.050214 |
| TTRNGHAA | 0.004312 | 0.643176 |
| KACDTWGN | 0.020031 | 0.035274 |
| TAAHYABW | -0.03625 | 0.000121 |
| ARMYAAGV | 0.019283 | 0.037902 |
| TGYGTACH | 0.015768 | 0.493004 |

| | | |
|---|---|---|
| TRTWTGCW | 0.018187 | 0.258766 |
| AAAMAAAR | -0.00935 | 0.024866 |
| ATMMAYAN | -0.01295 | 0.267248 |
| WTRTTTGY | -0.01487 | 0.272732 |
| TCRAYADW | 0.003956 | 0.751831 |
| GTACRCAH | -0.00138 | 0.965276 |
| TTYGCTYW | 0.015228 | 0.362641 |
| TNGCTHWG | 0.006644 | 0.521259 |
| TNACYMWG | -0.01242 | 0.160252 |

**Estimation of the binomial probability to observe a motif in at least n of N sequences of the analyzed set for random reasons**

The value of $P_{Bonf}(n,N)$ was calculated as follows. Consider a motif **M** = $m_1$, $m_2$, .., $m_k$ of length *k* written in the 15-letter IUPAC code. The probability of observing the motif **M** at a certain position of a DNA sequence can be estimated based on a Markov chain of order *r*:

$$P(M)=\frac{P(m_1,m_{r+1})P(m_2,m_{r+2})..P(m_{k-r},m_k)}{P(m_2,m_{r+1})P(m_3,m_{r+2})..P(m_{k-r},m_{k-1})}$$

$P(m_i,m_{i+r})$ is the frequency of occurrence of the motif $m_i$, $m_{i+1}$, .., $m_{i+r}$ in the set **Pos**. Alternatively, *P(M)* can be calculated assuming equal frequencies of the mononucleotide "background" context ($P_A = P_T = P_G = P_C = 0.25$).

Then, the probability *p* that a motif of length *k* occurs in a DNA sequence of length *L* at least once is

$$p=1-e^{-(L-k+1)*P(M)}$$

It may be noted that *Q* - expected representation of the motif in the **Pos** set is equal to *p*.

The statistical significance of observing the motif in at least *n* out of *N* sequences in the set Pos can be estimated by using the binomial criterion as

$$P_{one}(n,N)=\sum_{i=n}^{N}C_N^i p^i(1-p)^{N-i}$$

Because the number of motifs being tested for significance is large, we use the Bonferroni correction for multiple comparisons.

$$P_{Bonf}(n,N)=P_{one}(n,N)*15^k$$

**Assessment of the dependence of ChIP-seq peak scores in experiments with nine transcription factors on the presence of significant IUPAC motifs in their ChIP-seq peaks**

**CEBPA**

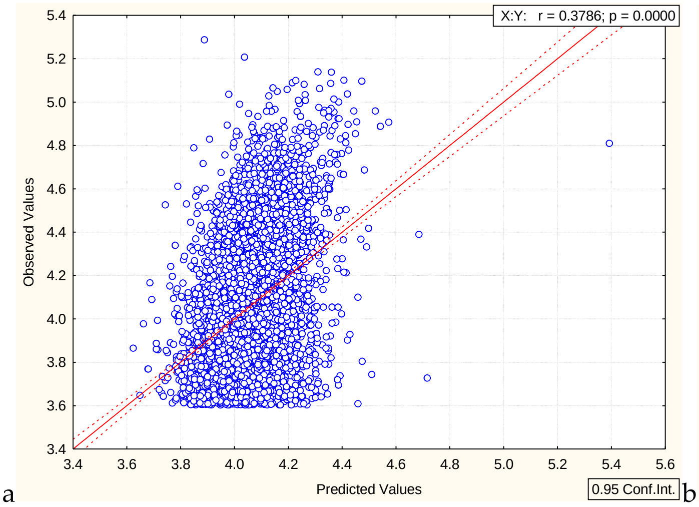


a

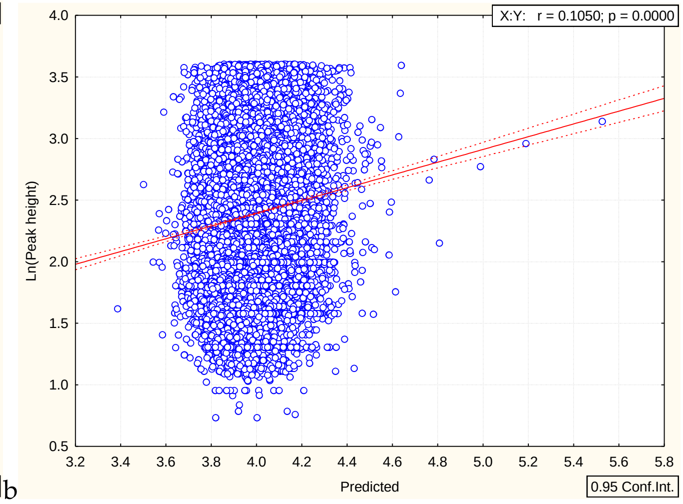


b

**CEBPB**

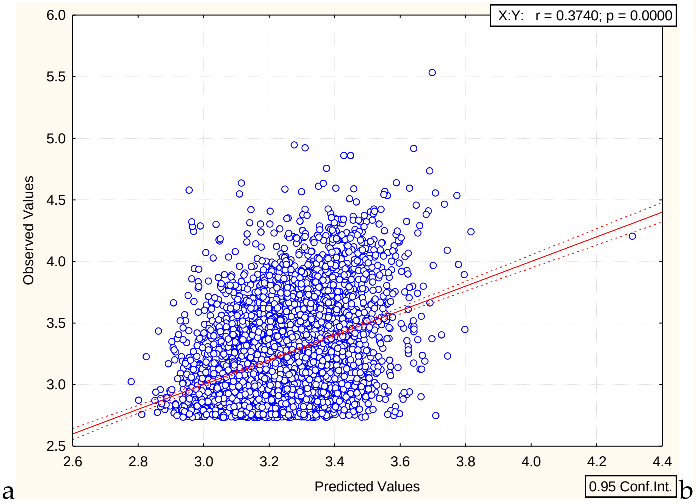


a

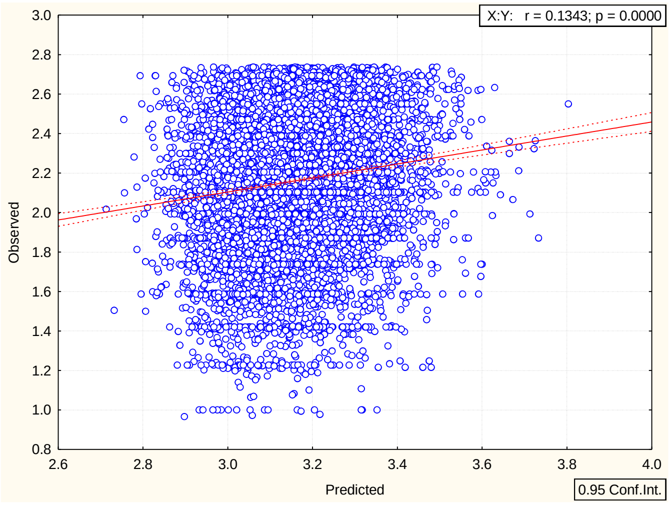


b

**NFE2L2**

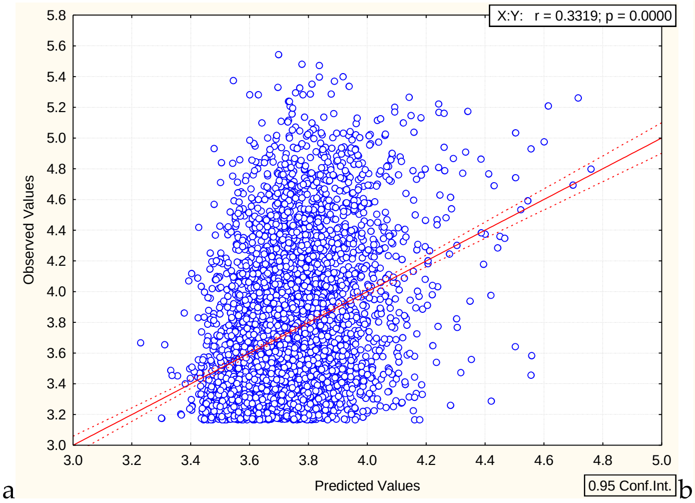


a

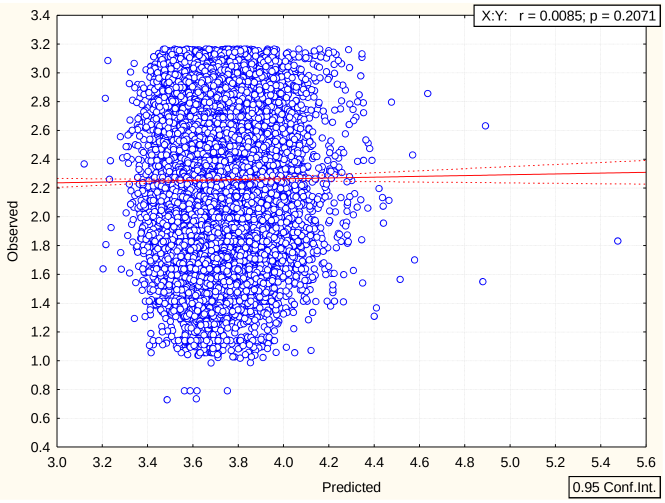


b

**SP1**

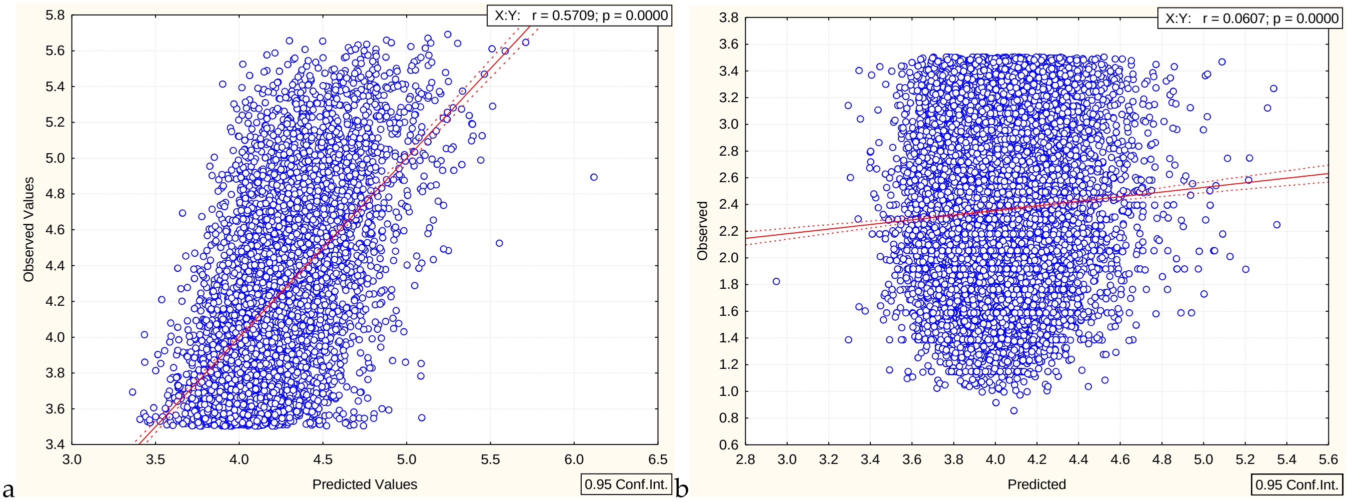


a b

**GATA1**

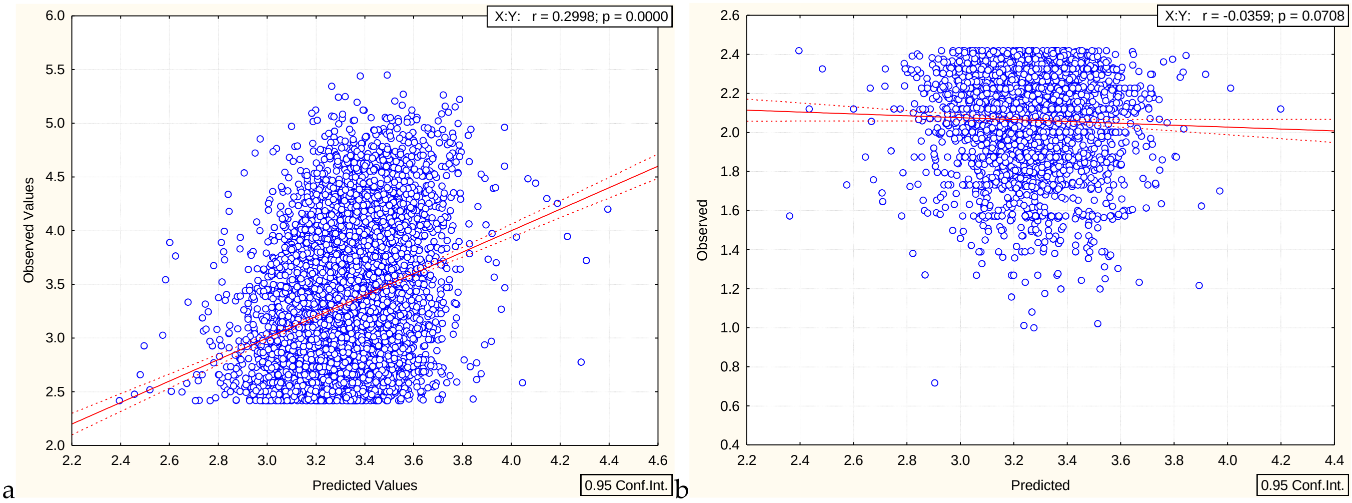


a b

**FOXO1**

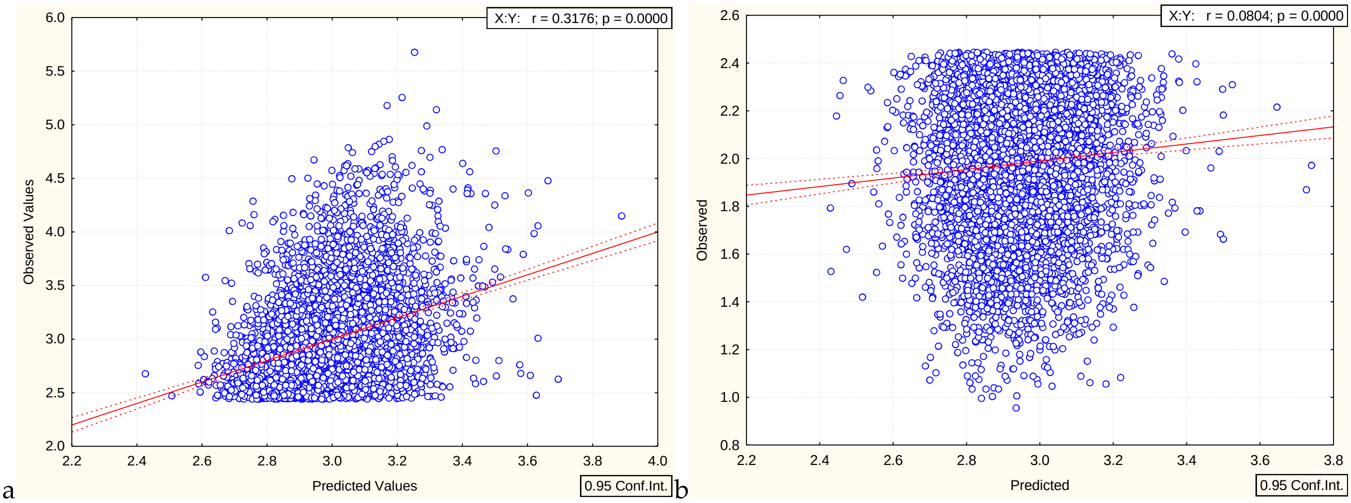


a b

**NFYA**

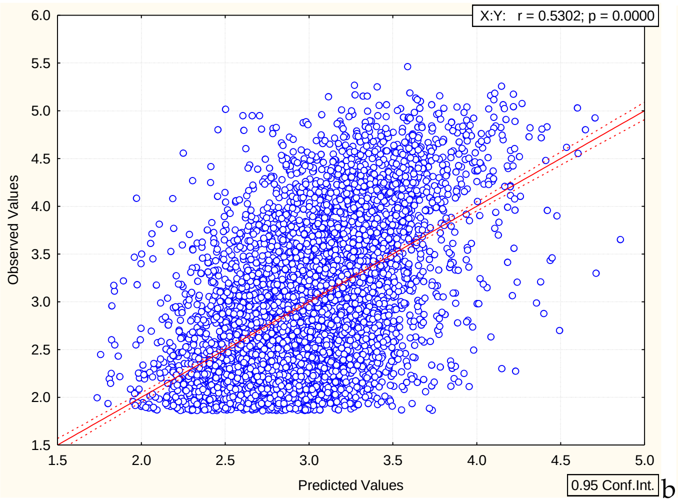


a

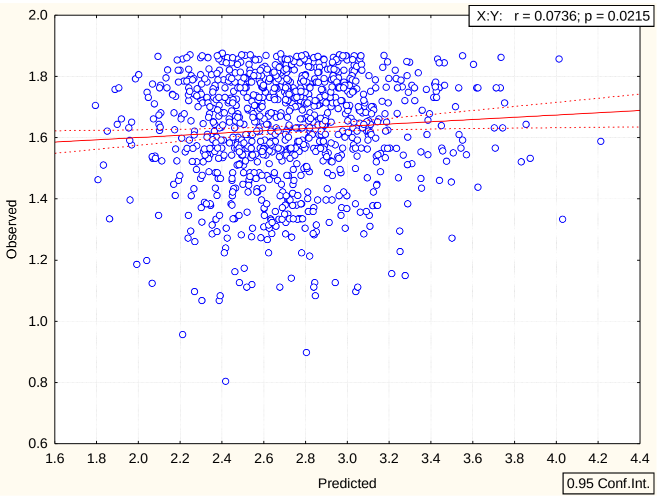


b

**MEF2D**

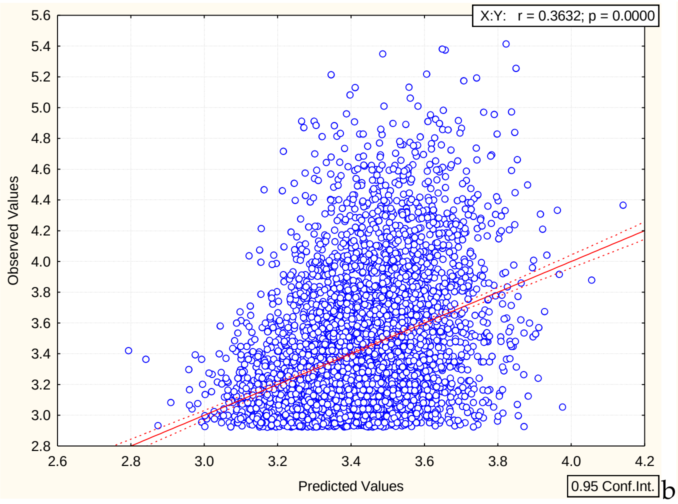


a

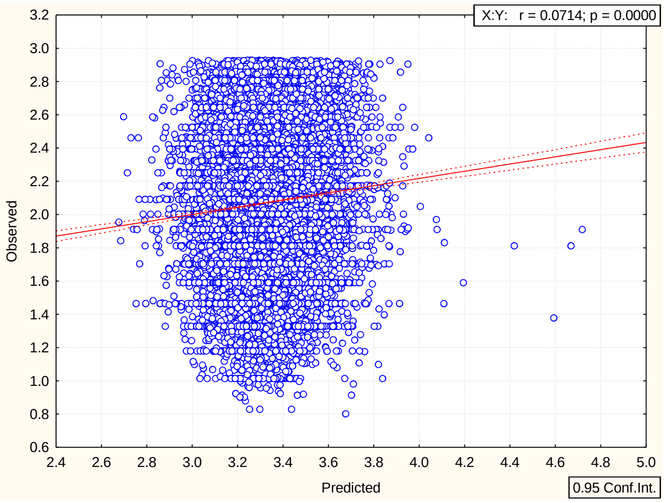


b

**STAT5B**

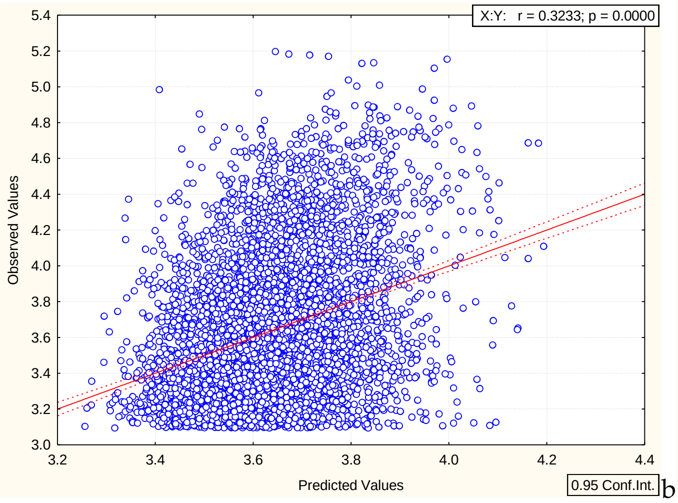


a

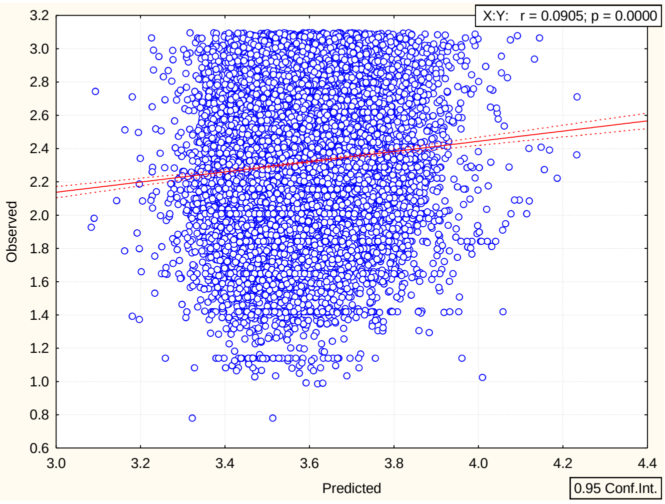


b

**Figure S1**. Dependence of the observed values of $ln(PS)_{Obs}$ for ChIP-seq peak sequences in the training (a) and control (b) sets on their expected value in experiments with nine transcription factors. Solid and dashed lines represent the regression line and the bounds of its 95% confidence interval as calculated using STATISTICA (StatSoft™, Tulsa, USA). *r* is the linear correlation coefficient and *p* is its statistical significance.

**Estimation of the influence of the boundary value $q_0$ on the number of IUPAC motifs considered**

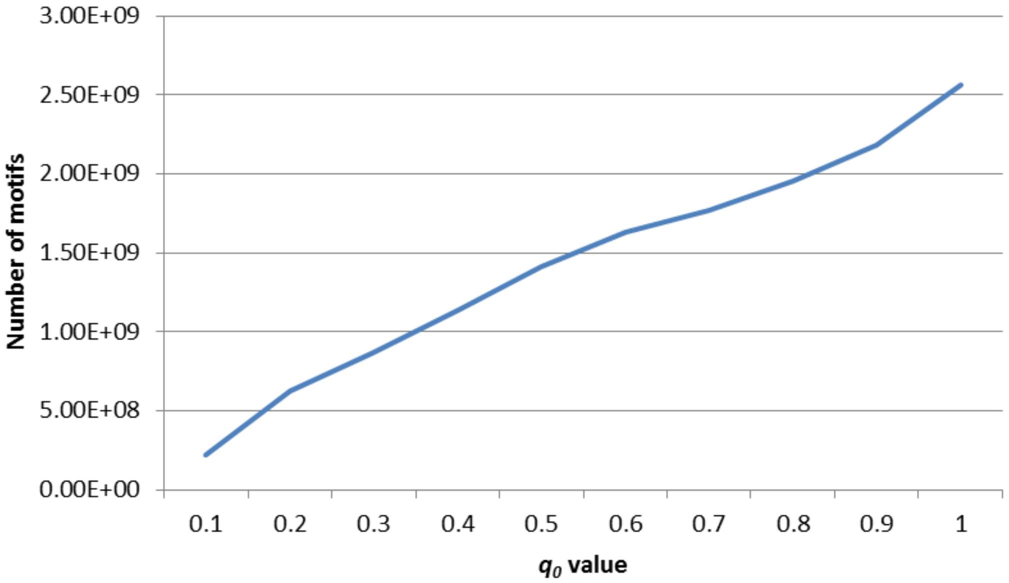


**Figure S2.** The dependence of the number of the motifs being considered ($y$-axis) on the boundary value $q_0$, the occurrence of the motifs by chance ($x$-axis). The search was carried out in a set of 1000 randomly generated 200-bp DNA sequences.
